# Nuclear-spin-pattern control of electron-spin dynamics in a series of V(iv) complexes†
†Electronic supplementary information (ESI) available: Methods, additional characterization and discussion. CCDC 1921675–1921677. For ESI and crystallographic data in CIF or other electronic format see DOI: 10.1039/c9sc02899d


**DOI:** 10.1039/c9sc02899d

**Published:** 2019-07-29

**Authors:** Cassidy E. Jackson, Chun-Yi Lin, Spencer H. Johnson, Johan van Tol, Joseph M. Zadrozny

**Affiliations:** a Department of Chemistry , Colorado State University , Fort Collins , CO 80523 , USA . Email: joe.zadrozny@colostate.edu; b National High Magnetic Field Laboratory , Tallahassee , FL 32310 , USA

## Abstract

Achieving control of phase memory relaxation times (*T*_m_) in metal ions is an important goal of molecular spintronics. Herein we provide the first evidence that nuclear-spin patterning in the ligand shell is an important handle to modulate *T*_m_ in metal ions. We synthesized and studied a series of five V(iv) complexes with brominated catecholate ligands, [V(C_6_H_4–*n*_Br_*n*_O_2_)_3_]^2–^ (*n* = 0, 1, 2, and 4), where the ^79/81^Br and ^1^H nuclear spins are arranged in different substitutional patterns. High-field, high-frequency (120 GHz) pulsed electron paramagnetic resonance spectroscopic analysis of this series reveals a pattern-dependent variation in *T*_m_ for the V(iv) ion. Notably, we show that it is possible for two molecules to have starkly different (by 50%) *T*_m_ values despite the same chemical composition. Nuclear magnetic resonance analyses of the protons on the ligand shell suggest that relative chemical shift (*δ*), controlled by the patterning of nuclear spins, is an important underlying design principle. Here, having multiple ligand-based protons with nearly identical chemical shift values in the ligand shell will, ultimately, engender a short *T*_m_ for the bound metal ion.

## Abstract 2

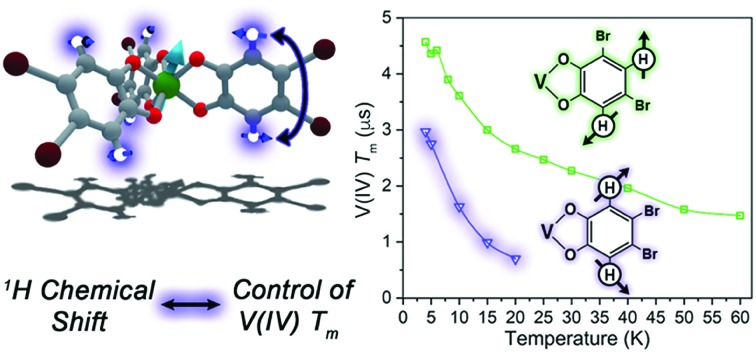
This report details how the design of specific nuclear-spin patterns on ligands modulates spin-relaxation times in a set of open-shell vanadium(iv) complexes.

## Introduction

Magnetic molecules are next-generation components of many different technological arenas, ranging from magnetic resonance imaging (MRI)^1^,^2^ to quantum information processing.^3^–^11^ Utility in any of these applications requires long spin-lattice relaxation times (*T*_1_ > 1 ms) and phase-memory relaxation times (*T*_m_§
§In this paper, we use *T*_m_ to describe the time constant associated with the decay of the echo intensity in a Hahn-echo experiment. This parameter is frequently also labelled *T*_2_, the spin–spin relaxation. However, the *T*_2_ designation is typically ascribed to relaxation in the *xy*-plane primarily from electronic spin flip-flop motions. In contrast, *T*_m_ is a broader, all-encompassing term for all relaxation processes that affect relaxation in the *xy*-plane. The *T*_m_ designation is particularly appropriate in this manuscript because there may be other factors controlling *T*_m_ (*e.g.* a short *T*_1_) under the experimental conditions. Further reading on this distinction can be found in ref. 12. > 100 μs). *T*_1_ defines the lifetime of an excited spin and is the upper limit of *T*_m_. In contrast, *T*_m_ is the lifetime of the electron spin superposition, or coherence time. Designing systems where both of these parameters are long is an acute challenge because of the ubiquitous spin bath (nearby electronic spins or nuclear spins), which produces a chaotic local magnetism that shortens *T*_1_ and *T*_m_ from spin–spin interactions.^12^–^15^ To circumvent the spin bath challenge, significant efforts are made to engineer environments with less noise from the spin bath. This engineering is done by dilution of the paramagnetic species, using smaller-magnetic-moment isotopic substitution (*e.g.*^2^H, *μ* = 0.86*μ*_N_ for ^1^H, *μ* = 2.79*μ*_N_), or complete elimination of nuclear spins.^3^,^16^–^20^ However, large-moment environmental spins are a critical part of utility in nearly all applications. For example, MRI applications require function in proton-rich biological environments, and information processing applications will likely feature stray magnetic fields from moving charges or other proximate magnetic materials in a device. Hence, understanding how to design complexes with long *T*_1_ and *T*_m_ in magnetic environments is a necessary advance for future technologies.

**Fig. 1:**
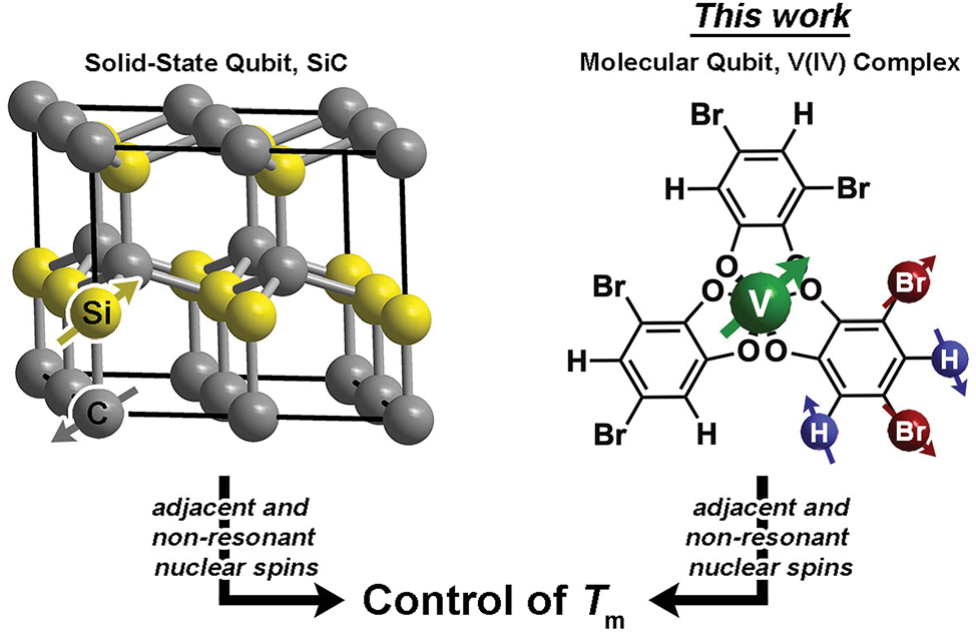
Defect qubits in SiC display a long *T*_m_ because only non-resonant spins can be adjacent in the structure, which disrupts nuclear spin diffusion. This manuscript tests whether such a design principle will affect *T*_m_ in molecular species, specifically by probing different patterns of ^1^H and ^79/81^Br spins on catecholate ligands in V(iv) complexes.

Herein, we demonstrate the first control of *T*_m_*via* patterning of ligand-based nuclear spins in a metal complex (Fig. 1). In molecules, ligand nuclear spins are a critical component of the nuclear spin bath and control electron spin dynamics through nuclear spin diffusion.^14^,^15^,^21^ In this phenomena, pairs of resonant nuclear spins (those that require identical quantities of energy to flip) engage in energy-conserving flip-flop motions, wherein two oppositely oriented spins simultaneously flip, or exchange spin.^22^ This process generates local magnetic noise and shortens *T*_m_ for a magnetic ion.^23^–^26^ In the absence of a nuclear spin bath, *T*_m_ will approach and exceed millisecond lifetimes.^27^–^30^ However, in spin-rich environments, lifetimes are typically less than 100 μs (ref. 31) and more frequently less than 10 μs.^19^,^23^,^32^–^52^

A recent breakthrough in the study of paramagnetic defects in SiC might hold the key to longer relaxation times in nuclear spin-rich baths (Fig. 1).^53^–^55^ The defects in SiC display longer *T*_m_ values than nitrogen vacancy centers in diamond despite higher nuclear spin concentration in the former (1.1% ^13^C and 4.7% ^29^Si in SiC; 1.1% ^13^C in diamond).^56^ The relative enhancement in *T*_m_ stems from a particular structural feature – each Si atom is surrounded by C and each C atom is surrounded by Si.^53^–^55^,^57^ This interstitial patterning increases *T*_m_ in two ways. First, two Si nuclear spins are never adjacent (likewise for C), and nuclear spin diffusion decreases for spins held far apart from one another.^12^ Second, the difference in the nuclear *g*_n_ factors for C and Si ensures that, even when adjacent, spin diffusion will not occur between the nonresonant ^13^C and ^29^Si nuclei. These results suggest specific positioning of nuclear spins as a potential method of *T*_m_ control *via* synthetic chemistry.

**Fig. 2:**
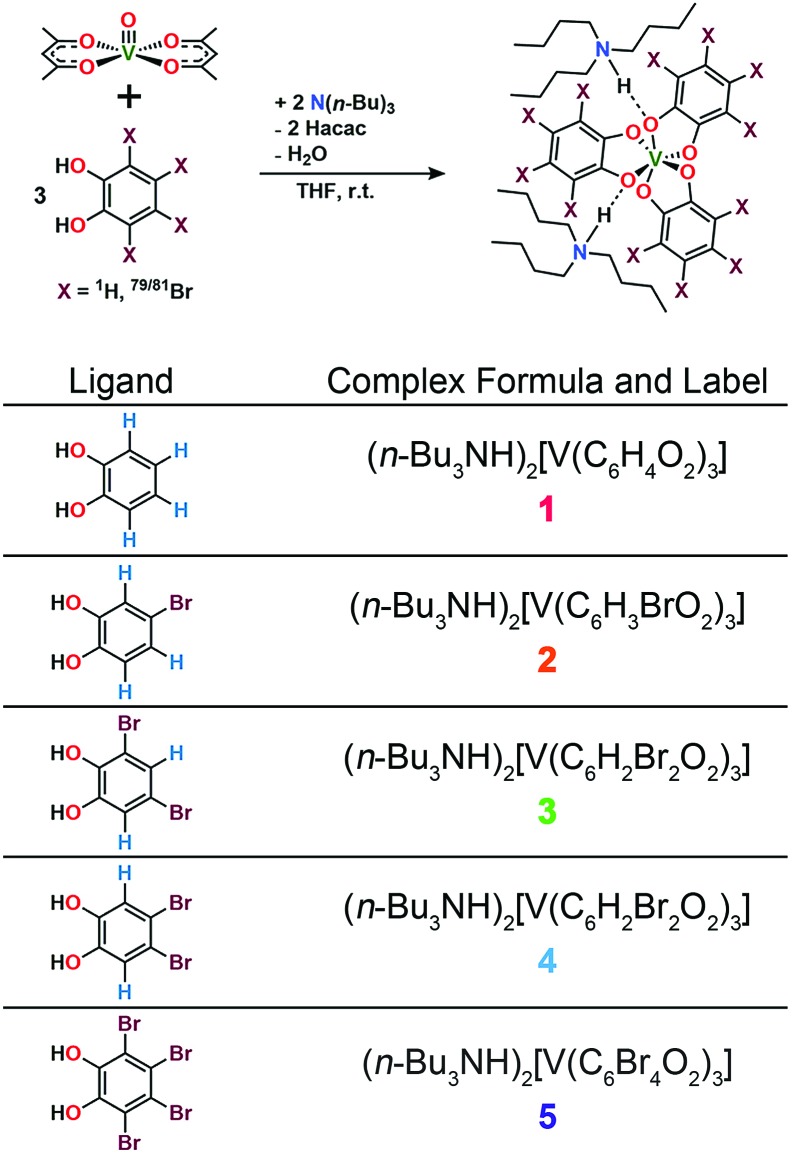
General synthetic scheme for **1–5** and labelling scheme for the studied complexes in this manuscript. See ESI† for additional synthetic information.

**Fig. 3:**
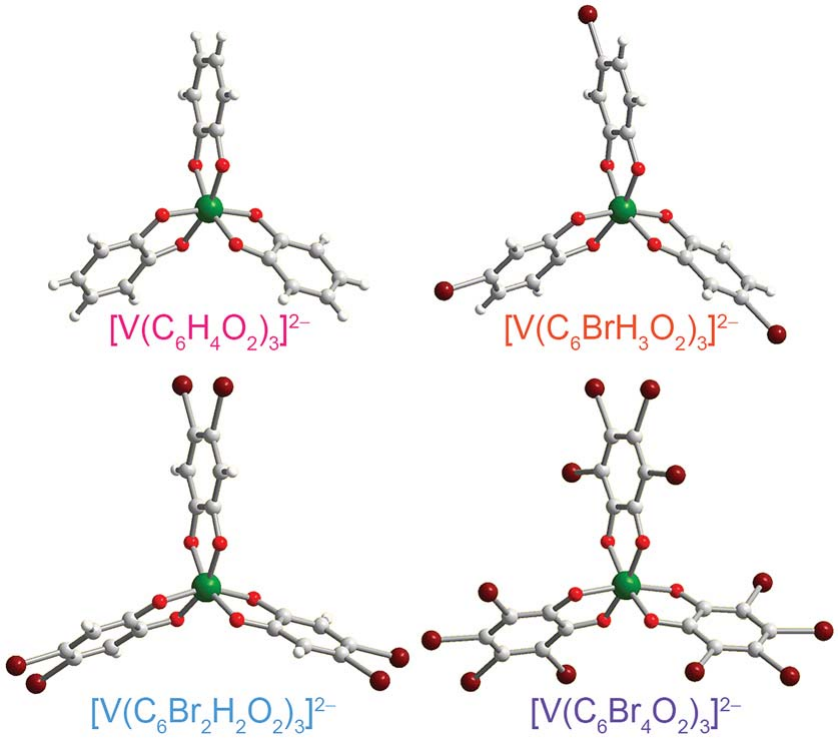
Molecular structures of the V(iv) complexes in **1** (ref. 40), **2**, **4**, and **5**, as determined from the crystal structures of these compounds. Counter ions are omitted for clarity. Green, maroon, red, gray, and white spheres represent vanadium, bromine, oxygen, carbon, and hydrogen atoms, respectively.

Drawing inspiration from SiC, we address the questions: can patterning of nuclear spins on ligand shells influence the electronic *T*_m_ of a ligated metal? Freedman and co-workers showed that separation between an open-shell ion and nuclear spins is important,^58^ and there is significant literature demonstrating the impacts on replacement of ^1^H (*μ* = 2.79*μ*_N_) with low-moment magnetic nuclei *e.g.*^2^H (*μ* = 0.86*μ*_N_).^19^,^42^,^46^ In this paper we probe a different question: can the impacts of high-magnetic-moment nuclei on *T*_m_ be mitigated instead by controlling nuclear spin to nuclear spin interactions in a molecule? To address these questions, we prepared and investigated, *via* pulsed EPR spectroscopy, a series of Bu_3_NH^+^ salts of the canonical tris(catecholato)vanadate(iv) complex [V(C_6_H_4_O_2_)_3_]^2–^: (*n*-Bu_3_NH)_2_[V(C_6_H_4_O_2_)_3_] (**1**), (*n*-Bu_3_NH)_2_[V(4-Br-C_6_H_3_O_2_)_3_] (**2**), (*n*-Bu_3_NH)_2_[V(3,5-Br_2_-C_6_H_2_O_2_)_3_] (**3**), (*n*-Bu_3_NH)_2_[V(4,5-Br_2_-C_6_H_2_O_2_)_3_] (**4**), and (*n*-Bu_3_NH)_2_[V(C_6_Br_4_O_2_)_3_] (**5**). In this series, the specific pattern of ^1^H and ^79/81^Br is varied on each ligand (Fig. 2 and 3). These nuclear spins have significantly different resonance frequencies,^56^ and, on this basis, we hypothesized that ligand-based nuclear-spin diffusion would be modulated in **1–5**. Furthermore, we predicted that such change would lead to a variation in V(iv) *T*_m_ dependent on the exact substitutional pattern of H and Br on the ligand. In this report, we show for the first time that this patterning design strategy is an effective means of influencing *T*_m_. Importantly, these studies also include the first investigation of the pulsed EPR spectroscopic properties of the V(iv) ion at very high field and frequency (>4.0 T, 120 GHz).

## Results and discussion

Preparation of the targeted complexes proceeded *via* a simple ligand metathesis scheme using VO(acac)_2_, tri-*n*-butylamine, and the ligands shown in Fig. 2 (see also ESI, Fig. S1–S4†). The general scheme for these syntheses follows previous reports of the triscatecholato complex of V(iv).^18^,^41^,^59^–^62^ Recrystallization afforded crystals suitable for single crystal X-ray diffraction of **2**, **4**, and **5** (but not **3**, see Fig. 3, Tables S1–S3, and ESI†). Single crystal X-ray diffraction experiments on crystals of **1**,^40^**2**, **4**, and **5** (Fig. 3) reveal similar molecular structures of the VO_6_ core. In these structures, all vanadium(iv) ions are in a six-coordinate environment, with average V–O bond lengths varying over a tight range across the series, from 1.938(4) Å for **1** to 1.943(9) Å for **5**. The continuous-shape-measurement (CSM) analyses using SHAPE 2.0 software^63^,^64^ of **1–5** provide symmetry measures for an octahedron of 1.67, 2.39, 3.97, and 1.41 for **1**, **2**, **4**, and **5**, respectively (here, a value of 0 corresponds to a perfect octahedron). The symmetry measures for a trigonal prismatic geometry are much higher (>5), indicating that **1**, **2**, **4**, and **5** are better described as slightly distorted octahedra (Table S4†). Beyond the first coordination sphere, all molecules exist hydrogen-bonded to two *n*-Bu_3_NH^+^ cations through the O atoms of the catecholate ligands (Fig. S5†). This interaction is similar across **1**, **2**, **4**, and **5**, with an average V···HNBu_3_^+^ distance of 2.99(6) Å. Prior experiments demonstrate that this association persists in solution (Fig. S5†).^40^

One critical aspect of the tested design principle relies on a difference between the spin-flip energies of the ^1^H and ^79/81^Br nuclear spins. On this merit, high-field and high-frequency EPR (120 GHz) was selected for studying **1–5**. With this technique, the differences in Larmor frequencies between ^1^H (*ca.* 187 MHz) and ^79/81^Br (*ca.* 47 and 51 MHz, respectively) at 4.4 T are large relative to those at the more conventional EPR frequency, X-band (0.4 T): ^1^H (*ca.* 17.0 MHz) and ^79/81^Br (*ca.* 4.3 and 4.6 MHz, respectively).^65^ Importantly, such conditions will decouple not only the ^1^H and ^79/81^Br nuclear spins, but potentially ^79^Br from ^81^Br due to the bigger difference in Larmor frequencies (*ca.* 4 MHz) – rendering the Br atoms inert spin blocks to disrupt ^1^H nuclear-spin diffusion. High-field, high-frequency investigations of the relaxation times of V(iv) are, to the best of our knowledge, unprecedented.

**Fig. 4:**
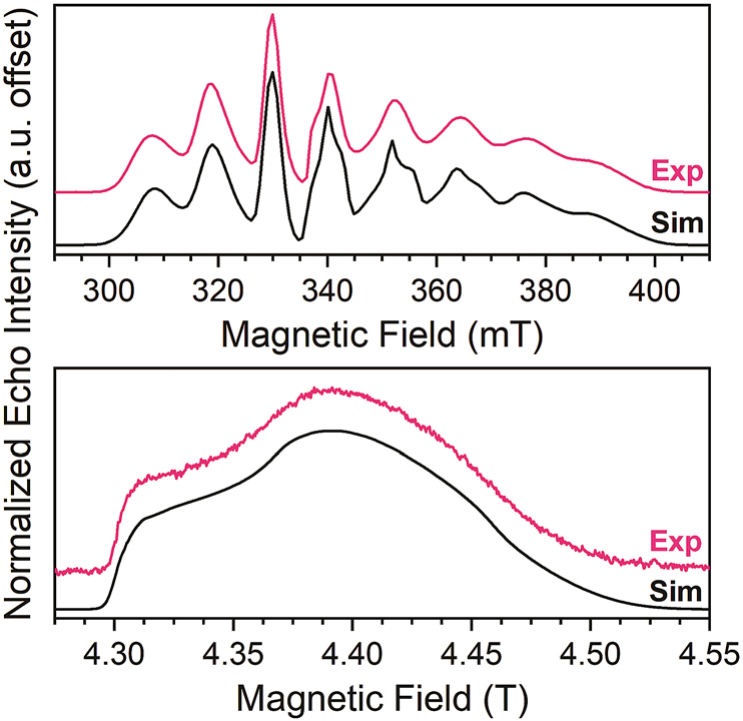
(Top) X-band (9.460 GHz) echo-detected, field-swept spectra of **1** (1 mM in OTP, color line) at 5 K and simulation (black). Data taken from ref. 40. (Bottom) 120 GHz echo-detected, field-swept spectra of 1 mM **1** in OTP solution (color) at 5 K and simulation (black).

Echo-detected EPR spectra of **1–5** were collected to test for variation in the spin-Hamiltonian parameters as a function of ligand. To do so, **1–5** were dissolved in d^14^-*o*-terphenyl (d^14^-OTP) at a 1 mM concentration, leveraging the solubility in nonpolar media afforded by the tri-*n*-butylammonium cations.^40^ The echo-detected, field-swept (EDFS) spectra of **1–5** were then collected at 5 K and 120 GHz (Fig. 4, S6 and Table S5†). The recorded spectra starkly differ in appearance from the 100 mT-wide, eight-line patterns observed at X-band frequency (Fig. 4). Instead, each 120 GHz spectrum reveals a single broad transition, spanning from 4.3–4.5 T. Such spectral width is attributed to enhanced broadening of the electronic *g*-factor (“*g*-strain”) at high magnetic fields.^15^ Simulations of the spectra were performed using Easyspin^66^ and the following spin Hamiltonian:***Ĥ*** = *g*_e_*μ*_B_***Bŝ*** – *g*_N_*μ*_N_***BÎ*** + ***ÎAŝ*** + ***ÎQÎ***Here, *g*_e_ and ***A*** correspond to rhombic electronic *g* factors and ^51^V hyperfine coupling constants, respectively. ***Q*** is the nuclear quadrupolar constant for ^51^V, ***ŝ*** and ***Î*** are electronic and nuclear spin operators, respectively, *μ*_B_ and *μ*_N_ are the Bohr and nuclear magnetons, respectively, *g*_n_ the nuclear *g* factor for ^51^V and ***B*** the magnetic field. More simulation details and the exact spin Hamiltonian values extracted can be found in the ESI and Table S4.† We note that the best simulations of the *g*-factors at 120 are only slightly different from those obtained at X-band.^40^,^41^ We place higher confidence on the *g*-factors determined here, since greater accuracy on this parameter is a hallmark of high frequency EPR.^15^,^67^–^69^ Most importantly, we note that the obtained parameters are similar in magnitude and anisotropy, demonstrating a relatively consistent electronic structure for the V(iv) ion in **1–5**. As the *g* and *A* values for V(iv) ions are also extremely sensitive to the symmetry of the ligand field,^70^ the similarity of these parameters highlights a relatively consistent local coordination geometry for **1–5** when frozen in d^14^-OTP.

**Fig. 5:**
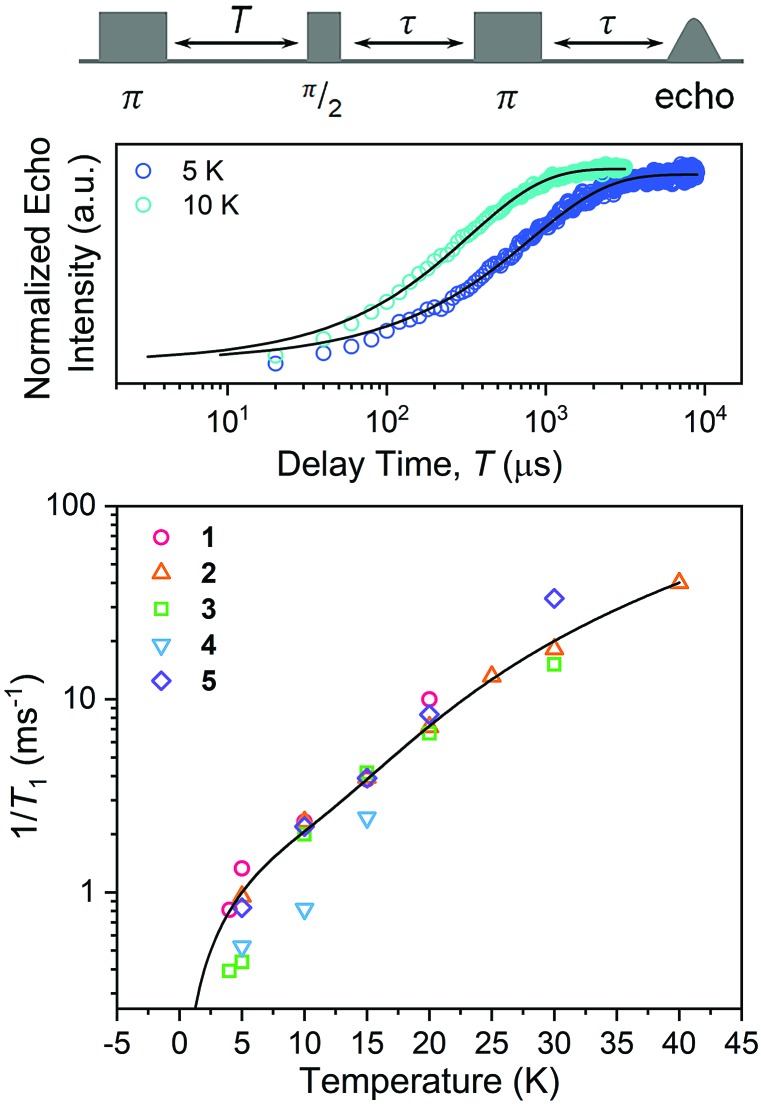
(Top) Selected variable temperature inversion recovery curves (color traces) and fits (black traces) for **1**. (Bottom) Variable-temperature 1/*T*_1_ data for **1–5**. Data were collected at 120 GHz frequency on samples of *ca.* 1 mM concentration in d^14^-OTP glass. The black trace is the fit to the direct and Raman processes. Error bars are generally under the width of the symbols – exact uncertainties are tabulated and depicted in the ESI.†

Spin-lattice relaxation rates (1/*T*_1_) were obtained as the first step toward understanding the impact of ligand nuclear-spin patterning on the V(iv) spin dynamics. To determine these rates for **1–5**, variable-temperature inversion recovery experiments were performed at the highest-intensity peaks in the 120 GHz EDFS spectra at 5 K, which is *B*_0_ = 4.4 T for all five complexes (Fig. 5, S7–S11 and Table S6†). For **1–5**, 1/*T*_1_ is slowest at low temperature, with an average 1/*T*_1_ of 0.88(6) ms^–1^ at 5 K. With increasing temperature, *T*_1_ rapidly decreases for **1–5**, in concert with a rapidly hastening relaxation rate, 1/*T*_1_. Owing to instrumental limitations related to the deadtime, performance of these experiments was precluded above 40 K. An immediate observation from these data is the near-two-orders-of-magnitude enhancement of 1/*T*_1_ (average 1/*T*_1_ = 0.88(6) ms^–1^) at 120 GHz *versus* the 5 K, 9.4 GHz 1/*T*_1_ of **1**: 0.0141(4) ms^–1^.^40^,^41^,^43^ Comparison of 1/*T*_1_ across the series of complexes, in contrast, reveals remarkable similarity between the temperature-dependent curves at this field/frequency.

The enhancement of spin-lattice relaxation rates (1/*T*_1_) at high field/frequency gives valuable information about the dominant high-field relaxation process for V(iv). Indeed, there are a collection of different mechanisms potentially responsible for spin-lattice relaxation: direct, Raman, local-mode, tunneling, and thermally activated processes.^15^,^71^ Yet, only direct, tunneling, and thermally activated processes are field-dependent. Of these, tunneling is typically suppressed under an applied field and thermally activated processes are likely precluded for V(iv) owing to the absence of low-lying excited states for this *S* = 1/2 ion. Hence, we hypothesized that a dominant direct process is responsible for the stark shortening of *T*_1_. To test this hypothesis, we modelled the temperature-dependence of the spin-lattice relaxation rate (1/*T*_1_) at 120 GHz. We found that the data for all complexes were readily modeled using the sum of a direct and Raman process in the following equation (see Fig. 5 and S12†):^15^
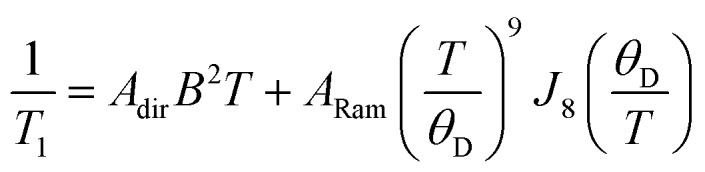
Here, *T* is temperature, *A*_dir_ is the direct process coefficient, *B* is the magnetic field, *A*_Ram_ is the Raman process coefficient, *θ*_D_ is the Debye temperature, *J*_8_(*θ*_D_/*T*) is the transport integral (see ESI† for full expression and resulting fit parameters). Qualitatively, because the Raman process is field-independent, the two-order-of-magnitude shortening of *T*_1_ at 120 GHz compared to 9.4 GHz is strongly suggestive of the direct process importance. Indeed, the two-order of magnitude difference in *T*_1_ between these two data sets would be expected from the *B*^2^ dependence of this process.^12^ Furthermore, we can successfully simulate the *T*_1_ data using the Raman process from X-band analyses and modulating only *A*_dir_, (see ESI†). Finally, we note that the shortening of *T*_1_ at the high fields of these analyses agrees with reported ac magnetic susceptibility studies.^41^,^43^ The most important observation, however, is that the relaxation mechanisms for **1–5** appear invariant with ligand identity.

**Fig. 6:**
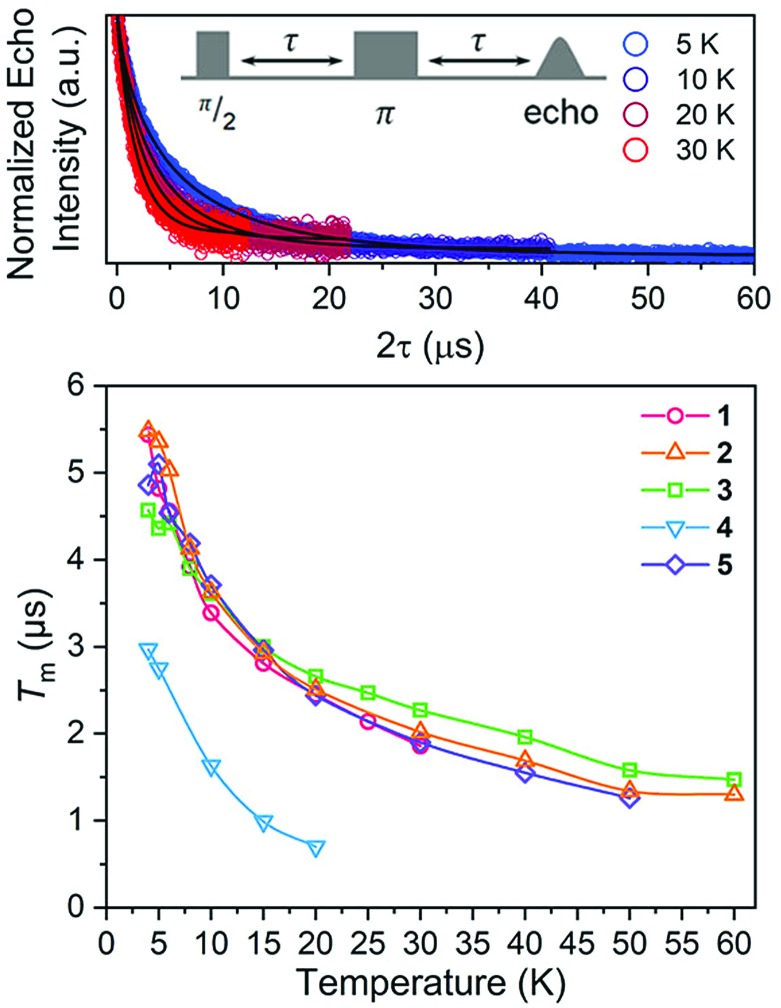
(Top) Selected variable temperature Hahn echo decay curves (color traces) and fits (black traces) for **1**. The pulse sequence is inset. (Bottom) Variable-temperature *T*_m_ data for **1–5**. Data were collected at 120 GHz frequency on samples of *ca.* 1 mM concentration *o*-terphenyl glass. Error values are generally within the width of the data symbols – exact uncertainties are tabulated and depicted in the ESI.†

Nuclear spin diffusion is expected to exert the greatest impact on *T*_m_, not *T*_1_.^3^,^12^,^21^,^23^ Hence, *T*_m_ was measured for **1–5** to test for a pattern-dependent effect. Variable-temperature, two-pulse Hahn echo experiments were performed on **1–5** in d^14^-OTP at 1 mM concentration to evaluate the echo decay as a function of ligand (Fig. 6 and S13–S17†). Stretched exponential functions were fit to these decays to extract *T*_m_ and the stretch parameter, *β*, (see ESI, Table S6†) which can give mechanistic insight into the decay of the superposition. All complexes display the longest *T*_m_s at the lowest temperatures. At 5 K, the *T*_m_ values of **1**, **2**, **3**, and **5** range from 4.36(8) to 5.36(9) μs. For **4**, *T*_m_ is about 2 μs shorter, 2.75(3) μs. With increasing temperatures, *T*_m_ drops sharply for **1–5**. By 20 K, the *T*_m_ of **4** is 0.7(1) μs, which precluded pulsed measurements at any higher temperatures owing to low signal to noise. In contrast, *T*_m_ remains appreciable for **1–3** and **5** up to at least 30 K. Interestingly, above 20 K, *T*_m_ for **3** is slightly higher than the other complexes. The stretch parameters increase with increasing temperatures for **1–5**. For **1–3** and **5**, *β* ranges over 0.6 to 0.8 at the lowest temperatures, and venture closer to 1 at the highest temperatures. For **4**, however, *β* is close to 1 at the lowest temperature and only increases with higher temperatures.

The difference in *T*_m_ for **4** relative to **3**, and **3**, **4** relative to **1**, **2**, and **5**, represent two significant findings. First, these data, particularly for **3** (*T*_m_ = 4.36(8) μs) and **4** (*T*_m_ = 2.75(3) μs), reveal for the first time that two substitutional isomers of the same magnetic metal complex can have significantly different *T*_m_ values. Second, the data for **3***versus* the rest of the series highlight the possibility of enhancing *T*_m_*via* patterns that avoid two adjacent protons (though that impact is modest in the present system). However, in that context, it is particularly puzzling that **4** demonstrates a significantly shorter *T*_m_ than **1** and **5**, which possess the highest number of adjacent ^1^H and ^79/81^Br spins, respectively. It is further puzzling that **4** displays a shorter *T*_m_ than **3**, when the protons in **4** are clearly further separated than those of **3**. Changes in geometry of the coordination site and electronic structure are powerful mechanisms for adjusting *T*_m_.^12^ However, the similarity of the spin-Hamiltonian parameters, crystallographic data, and *T*_1_ values for **1–5** suggests that the different substitutional patterns of the ligands do not affect these aspects of the V(iv) ion. These results thus suggest that the impact of the pattern on *T*_m_ is truly magnetic in nature, stemming from the ^1^H and ^79/81^Br ligand nuclear spins (*vide infra*).

**Fig. 7:**
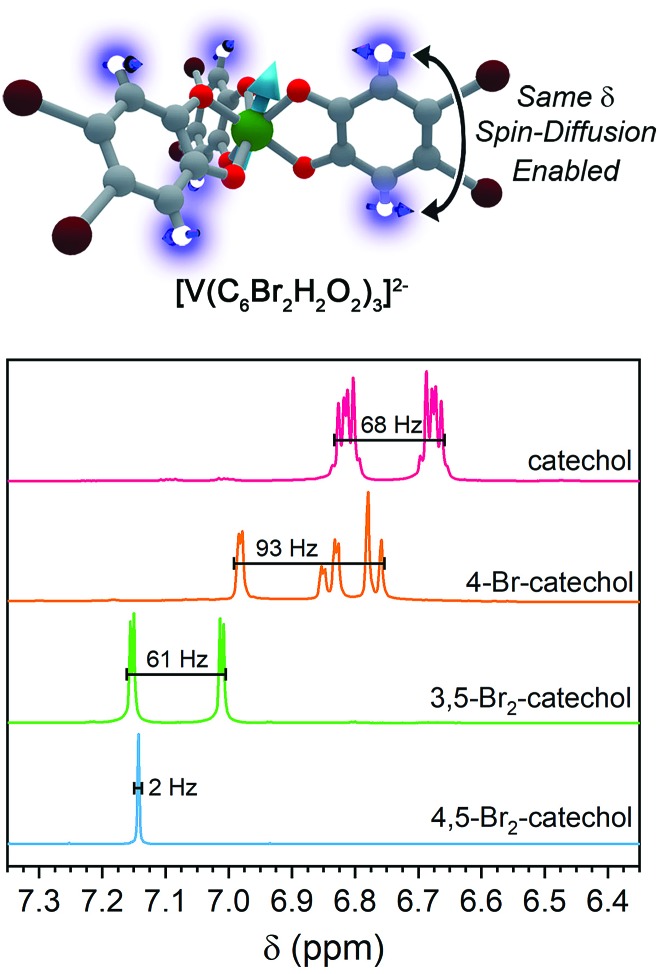
(Top) Hypothesized impact of patterning in the anomalously short *T*_m_ for **4**. (Bottom) Solution-phase, 400 MHz NMR spectra for the ligands in complexes **1–4**, focused explicitly on the aromatic region of chemical shift (*δ*). The relatively small spread in ^1^H NMR frequencies for 4,5-dibromocatechol relative to the ^1^H signals on the other ligands ensures that the protons in **4** always have a relatively proximate, resonant-spin neighbor. Hence, we hypothesize that these nuclei readily participate in nuclear spin diffusion and shorten *T*_m_ for the V(iv) ion.

For ligand-based nuclear spin diffusion to operate efficiently, the nuclear spins should have the same resonant NMR frequency (chemical shift). To probe the environment of nuclear spins for the molecules in this paper, we collected the ^1^H NMR spectra of the ligands.^3^,^15^ The 400 MHz (= 9.4 T field) ^1^H NMR spectra of the ligands of **1**, **2**, and **3** demonstrate peaks of varying multiplicity over a range of chemical shifts (in frequency, 68, 93, and 61 Hz, respectively) (Fig. 7). The observed range of ^1^H chemical shifts in the ligands of **1–3** is a consequence of two factors. First, the presence of different quantities and arrangements of bromine on the ligands adjust the ^1^H chemical shifts. Second, *J*-coupling between the aromatic ^1^H protons split the individual ^1^H peaks into multiplets. In contrast, the aromatic protons of 4,5-dibromocatechol (the ligand of **4**) yield a single peak with a full-width half-maximum of 2 Hz. The protons in this ligand are constrained to this tight chemical-shift window by the two-fold rotational symmetry and weak *J*-coupling (0–1 Hz) for aromatic 3,6 protons.^72^,^73^

On the basis of the NMR data, we rationalize the observed *T*_m_ data in terms of synthetic control *via* ligand-shell nuclear spin diffusion. First, the high symmetry of the 4,5-dibromocatechol and weak *J*-coupling ensure nearly identical chemical shifts for the aromatic ^1^H nuclei. In the other ligands, in contrast, asymmetry and stronger ^1^H–^1^H *J*-coupling spread the ^1^H chemical shifts out over >60 Hz. Owing to the relatively tight range (2 Hz) of the ^1^H frequencies in the ligand of **4** relative to **1–3**, we posit that a given ligand proton in **4** is significantly more likely to have a proximate nucleus (across the aromatic ring) with the same chemical shift. Hence, the ligand-based protons in **4** are more likely to engage in nuclear spin diffusion and impact *T*_m_. In the other complexes, *J*-coupling and differing chemical shifts spread the spin-flip frequencies of the ^1^H protons over a wider range, ensuring a lower probability that a given ^1^H will have a neighbor with precisely the same chemical shift. Hence, **1–3**, with non-resonant ^1^H spins, would be expected to display less-efficient spin diffusion and show a longer *T*_m_. This effect may also be operative for the ligand bromines, due to the different isotopes of bromine (^79^Br and ^81^Br). Hence, this argument may explain why **5** has a *T*_m_ in the same range of **1–3**. We note that the stretch parameters *β* are slightly higher for **4** relative to **1–3** and **5**, suggesting that nuclear spin-diffusion may be more operative for **4** (and consistent with our rationale).^21^ However, the typical values of *β* for dominant nuclear-spin diffusion are closer to 2–2.5,^12^ and this discrepancy may be related to the limitation of *T*_m_ by the short *T*_1_ in **1–5**. Given the fast spin-lattice relaxation at high field, lower-field measurements may engender stronger nuclear-spin-diffusion control by pushing ligand-based nuclear spin resonant frequencies closer together.^69^,^74^–^77^ Such measurements are exciting next studies.

## Conclusions and outlook

The foregoing results demonstrate, for the first time, that control of phase memory relaxation times is possible *via* nuclear-spin patterning within a molecule. Importantly, we interpret our data to suggest that tuning relative chemical shifts, which are dictated by the symmetry and chemical make-up of the molecule, are a key future design strategy for manipulating *T*_m_ in magnetic complexes. However, multiple new avenues of work are necessary to fully test the presented design strategy. In particular, learning how to harness said strategy to improve *T*_m_ is a pressing concern. Indeed, we note that the “optimally patterned” species **3** only exhibits a slight enhancement of *T*_m_ over a fully-protonated complex and the most dramatic impact is a shortening, not lengthening of *T*_m_. Toward the understanding to use this mechanism to lengthen *T*_m_, our future work spans studying the nuclear spin dynamics (in particular, the time constants for spin diffusion and spin–spin relaxation) of the ligands and metal complexes. In this context, an important absence in the above analyses is a direct picture of the spin dynamics of the ^79/81^Br nuclear spins, which is extremely challenging to obtain with solution-phase NMR. A system patterned with ^19^F nuclei is in contrast particularly advantageous since ^19^F NMR is readily performed.^78^–^80^ It is in these directions that we are now working.

## Conflicts of interest

There are no conflicts to declare.

## Supplementary Material

Supplementary information

Crystal structure data
