# Supplementary material for: Nuclear-spin-pattern control of electron-spin dynamics in a series of V(iv) complexes
Source: Chem Sci. 2019 Jul 29;10(36):8447–54. doi: 10.1039/c9sc02899d (PMC6839508; doi:10.1039/c9sc02899d)
Supplement: Supplementary file 1 [file SC-010-C9SC02899D-s001.pdf]

Electronic Supplementary Material (ESI) for Chemical Science.
This journal is © The Royal Society of Chemistry 2019

Electronic Supplementary Information for:

Nuclear-Spin-Pattern Control of Electron-Spin Dynamics in a Series of V(IV) Complexes

Cassidy E. Jackson,[1] Chun-Yi Lin,[1] Spencer H. Johnson,[1] Johan van Tol,[2] and Joseph M. Zadrozny*[1]

[1]*Department of Chemistry, Colorado State University, Fort Collins, Colorado 80523, USA.*
[2]*National High Magnetic Field Laboratory, Tallahassee, Florida 32310, USA.*

Chem. Sci.

Table of Contents

Description		Page
**Full Experimental Details.**		**S3**
**Table S1.**	Crystallographic information for the structural refinement of **2**.	**S13**
**Table S2.**	Crystallographic information for the structural refinement of **4**.	**S14**
**Table S3.**	Crystallographic information for the structural refinement of **5**.	**S15**
**Table S4.**	Continuous-shape-measurement (CSM) analysis results for **1**–**3** and **5**.	**S16**
**Table S5.**	Spin Hamiltonian parameters at 120 GHz for **1**−**5** at 5 K.	**S17**
**Table S6.**	Fit T_1 values and spectral diffusion parameters q.	**S18**
**Table S7.**	Fit parameters for the temperature dependence of T_1.	**S19**
**Table S8.**	Fit T_m values and stretch parameters β.	**S20**
**Fig. S1.**	^{1}H NMR spectrum of **2** in $CDCl_3$.	**S21**
**Fig. S2.**	^{1}H NMR spectrum of **3** in $CDCl_3$.	**S21**
**Fig. S3.**	^{1}H NMR spectrum of **4** in $CDCl_3$.	**S22**
**Fig. S4.**	^{1}H NMR spectrum of **5** in $CDCl_3$.	**S22**
**Fig. S5.**	Depiction of hydrogen bonding interactions in **1**, **2**, **4**, and **5**.	**S23**
**Fig. S6.**	120 GHz EDFS spectrum of **1**−**5** in d^{14}-*o*-terphenyl at 5 K.	**S24**
**Fig. S7.**	Selected variable-temperature inversion recovery curves and fits for **1**.	**S25**
**Fig. S8.**	Selected variable-temperature inversion recovery curves and fits for **2**.	**S26**
**Fig. S9.**	Selected variable-temperature inversion recovery curves and fits for **3**.	**S27**
**Fig. S10.**	Selected variable-temperature inversion recovery curves and fits for **4**.	**S28**
**Fig. S11.**	Selected variable-temperature inversion recovery curves and fits for **5**.	**S29**
**Fig. S12.**	T_1 fits showing the direct process, Raman processes, and sum.	**S30**
**Fig. S13.**	Selected variable-temperature Hahn echo decay curve and fits for **1**.	**S31**
**Fig. S14.**	Selected variable-temperature Hahn echo decay curve and fits for **2**.	**S32**
**Fig. S15.**	Selected variable-temperature Hahn echo decay curve and fits for **3**.	**S33**
**Fig. S16.**	Selected variable-temperature Hahn echo decay curve and fits for **4**.	**S34**
**Fig. S17.**	Selected variable-temperature Hahn echo decay curve and fits for **5**.	**S35**
**Fig. S18.**	Variable-temperature T_1 and T_m plot for **1**−**5**.	**S36**
**Fig. S19.**	Variable-temperature T_1 and T_m plot for **1**−**5** with error bars depicted.	**S37**
**Fig. S20.**	Deconvoluted X-band and 120 GHz EPR spectra for **1**.	**S38**
**References**		**S39**

Full Experimental Details

General Considerations. The complex (n-$Bu_3NH)_2[V(C_6H_4O_2)_3]$ (**1**) was reported to be air-sensitive.[1] Thus, all manipulations and syntheses of it and **2–5** were performed under a N_2 atmosphere with either a Vigor glovebox or Schlenk techniques. Glassware was either oven-dried at 150 °C for at least 4 h and/or flame-dried before bringing into the glovebox. Tetrahydrofuran (THF), diethyl ether (Et_2O), and hexanes were dried using a commercial solvent purification system from LC Technology Solutions and were stored over 4 Å molecular sieves prior to use. 4 Å molecular sieves were stored in a 150 °C oven and were activated at 280 °C under reduced pressure for at least 12 h prior to use. THF, Et_2O, and hexanes were subjected to a test with a standard purple solution of sodium benzophenone ketyl in THF to confirm low O_2 and H_2O content. Tri-*n*-butylamine (n-Bu_3N) and hexamethyldisiloxane (HMDSO) were purchased from commercial suppliers and dried by stirring over CaH_2 for at least 24 h then distilled over CaH_2. Tetrabromocatechol, 4-bromocatechol, and d^{14}-*o*-terphenyl were purchased from commercial suppliers and were used as received. Vanadyl acetylacetonate ($VO(acac)_2$),[2] 3,5-dibromocatechol,[3] 4,5-dibromocatechol,[4] and (n-$Bu_3NH)_2[V(C_6H_4O_2)_3]$ (**1**)[1,5] were prepared following the literature procedures.

(n-$Bu_3NH)_2[V(4$-Br-$C_6H_3O_2)_3]$ (2) In a N_2-filled glovebox, a 20-mL scintillation vial was charged with 0.135 g (0.509 mmol) $VO(acac)_2$, 0.283 g (1.49 mmol) 4-bromocatechol, ca. 8 mL THF, and a Teflon-coated magnetic stir bar. A solution of 238 μL (1.00 mmol) n-Bu_3N was added to the mixture with gentle shaking, resulting almost immediately in an intensely dark blue solution. The reaction mixture was allowed to stir overnight. All volatile materials (THF, H_2O, and acetylacetone) were removed under reduced pressure. The resulting dark blue residue was washed twice with ca. 5 mL cold Et_2O (Note: the complex is slightly soluble in Et_2O) and twice with ca. 5

mL hexanes and then was dried under reduced pressure to yield 0.456 g of dark blue powder. The solid was further purified by slow evaporation of THF in the glovebox for 2 weeks to afford 0.472 g (0.479 mmol, 96 % yield) of large, shiny, dark blue crystals suitable for single-crystal X-ray diffraction. The ^{1}H NMR spectrum of **2** in $CDCl_3$ reveals extremely broadened signals that are depicted in Fig. S2. IR (KBr, cm^{-1}): 3086, 2959, 2928, 2874, 2775, 2673, 2608, 2555, 1590, 1555, 1537, 1510, 1467, 1402, 1353, 1347, 1289, 1288, 1261, 1213, 1197, 1116, 1070, 1047, 1023, 927, 892, 847, 795, 736, 658, 633, 578, 563, 517, 464, 442, 415. UV-vis (THF); λ_{max} (ε_M, $M^{-1}cm^{-1}$): 293 (16000); 462 (3400); 576 (5300); 682 (4800). LTQ-MS (m/z): positive ion mode: $\{(n\text{-}Bu_3NH)_3[V(C_6H_3BrO_2)_3]\}^+$, 1170.08; $\{(n\text{-}Bu_3NH)_2[HV(C_6H_3BrO_2)_3]\}^+$ (MeCN), 1024.00; negative ion mode: $\{(n\text{-}Bu_3NH)(n\text{-}Bu_3N)[HV(C_6H_3BrO_2)_3]\}^-$ (THF), 1291.58; $\{[V(C_6H_3BrO_2)_3]\}^-$, 612.91; $\{[VO(C_6H_3BrO_2)_2]\}^-$, 441.00; $\{[V(C_6H_3BrO_2)_2]\}^-$, 425.16. Combustion analyses calculated for $C_{42}H_{65}Br_3N_2O_6V$ (found): 51.21 (51.23) % C; 6.57 (6.65) % H; 2.27 (2.85) % N.

$(n\text{-}Bu_3NH)_2[V(3,5\text{-}Br_2\text{-}C_6H_2O_2)_3]$ (3) In a N_2-filled glovebox, a 20-mL scintillation vial was charged with 0.133 g (0.502 mmol) $VO(acac)_2$, 0.411 g (1.53 mmol) 3,5-dibromocatechol, ca. 8 mL THF, and a Teflon-coated magnetic stir bar. A solution of 238 μL (1.00 mmol) *n*-Bu_3N was added to the mixture with gentle shaking, resulting almost immediately in an intensely dark blue solution. The reaction mixture was allowed to stir overnight. All volatile materials (THF, H2O, and acetylacetone) were removed under reduced pressure. The resulting dark blue residue was washed twice with ca. 5 mL cold Et_2O (Note: the complex is slightly soluble in Et_2O) and twice with ca. 5 mL hexanes and then was dried under reduced pressure to yield 0.518 g (0.424 mmol, 84 % yield) of pure dark blue powder indicated by elemental analysis. All crystallization attempts resulted in samples where crystals were too small for diffraction or amorphous solids. We

hypothesize that crystals of high quality for **3** were unable to be obtained presumably due to extensive disorder of the three asymmetric 3,5-dibromocatecholate ligands on the V(IV) ion. The ^{1}H NMR spectrum of **3** in $CDCl_3$ reveals extremely broadened signals that are depicted in Fig. S3. IR (KBr, cm^{-1}): 3068, 2960, 2931, 2872, 1583, 1526, 1465, 1419, 1373, 1357, 1345, 1329, 1287, 1252, 1234, 1227, 1187, 1101, 1074, 1059, 995, 943, 923, 828, 798, 789, 754, 732, 716, 708, 668, 658, 624, 578, 536, 464. UV-vis (THF); λ_{max} (ε_M, $M^{-1}cm^{-1}$): 293 (15000); 391 (5000); 580 (8100); 682 (7200). LTQ-MS (m/z): positive ion mode: $\{[n\text{-}Bu_3NH][C_6H_4Br_2O_2]\}^+$, 453.33; negative ion mode: $\{[V(C_6H_2Br_2O_2)_2]\}^-$, 577.00; $\{[VO(C_6H_2Br_2O_2)]\}^-$, 328.91. Combustion analyses calculated for $C_{42}H_{62}Br_6N_2O_6V$ (found): 41.58 (41.30) % C; 5.44 (5.12) % H; 2.18 (2.29) % N.

(n-Bu$_3$NH)$_2$[V(4,5-Br$_2$-C$_6$H$_2$O$_2$)$_3$] (4) In a N_2-filled glovebox, a 20-mL scintillation vial was charged with 0.133 g (0.502 mmol) $VO(acac)_2$, 0.411 g (1.53 mmol) 4,5-dibromocatechol, ca. 8 mL THF, and a Teflon-coated magnetic stir bar. A solution of 238 μL (1.00 mmol) n-Bu_3N was added to the mixture with gentle shaking, resulting almost immediately in an intensely dark blue solution. The reaction mixture was allowed to stir overnight. All volatile materials (THF, H_2O, and acetylacetone) were removed under reduced pressure. The resulting dark blue residue was washed twice with ca. 5 mL cold Et_2O (Note: the complex is slightly soluble in Et_2O) and twice with ca. 5 mL hexanes and then was dried under reduced pressure to yield 0.556 g of dark blue powder. The solid was further purified by slow evaporation of THF in the glovebox for 2 weeks to afford 0.405 g (0.332 mmol, 66 % yield) of large, shiny, dark blue crystals suitable for single-crystal X-ray diffraction. The ^{1}H NMR spectrum of **4** in $CDCl_3$ reveals extremely broadened signals that are depicted in Fig. S4. IR (KBr, cm^{-1}): 3094, 2961, 2933, 2872, 2746, 2659, 2540, 1556, 1536, 1493, 1462, 1355, 1337, 1257, 1226, 1186, 1080, 1026, 919, 873, 850, 836, 802, 734, 674, 656, 602, 535, 472, 412, 408. UV-vis (THF); λ_{max} (ε_M, $M^{-1}cm^{-1}$): 293 (11000); 579 (5100);

682 (45000). LTQ-MS (m/z): positive ion mode: {(*n*-$Bu_3NH)_3[V(C_6H_2Br_2O_2)]\}^+$, 1406.92; {[n-$Bu_3NH][C_6H_2Br_2O_2]\}^+$, 453.33; negative ion mode: $\{[V(C_6H_2Br_2O_2)_3]\}^-$, 849.50; $\{[V(C_6H_2Br_2O_2)_2]\}^-$, 582.92; $\{[VO(C_6H_2Br_2O_2)_2]\}^-$, 598.75; $\{[VO(C_6H_2Br_2O_2)]\}^-$, 328.91. Combustion analyses calculated for $C_{42}H_{62}Br_6N_2O_6V$ (found): 41.66 (41.30) % C; 5.24 (5.12) % H; 2.29 (2.29) % N.

(*n*-$Bu_3NH)_2[V(C_6Br_4O_2)_3]$ (5) In a N_2-filled glovebox, a 20-mL scintillation vial was charged with 0.0921 g (0.347 mmol) $VO(acac)_2$, 0.451 g (1.06 mmol) tetrabromocatechol, and a Teflon-coated magnetic stir bar. A solution containing 0.169 g (0.912 mmol) *n*-Bu_3N in 5 mL THF was added to the mixture with gentle shaking, resulting almost immediately in an intensely dark blue solution. The reaction mixture was allowed to stir overnight. All volatile materials (THF, H_2O, and acetylacetone) were removed under reduced pressure. The resulting dark blue residue was washed twice with ca. 5 mL Et_2O and then was dried under reduced pressure to yield 0.53 g of dark blue powder. The solid was further purified by crystallization with HMDSO/THF in a −35 °C freezer in the glovebox for 2−3 days to afford 0.451 g (0.266 mmol, 76.6 % yield) of large, shiny, dark blue crystals suitable for single-crystal X-ray diffraction (See Figure S21 for picture). The ^1H NMR spectrum of **5** in $CDCl_3$ reveals extremely broadened signals that are depicted in Fig. S2. IR (KBr, cm^{-1}): 3048, 2956, 2872, 1568, 1465, 1377, 1253, 1097, 1014, 870, 799, 733, 628, 537, 501, 412. UV-vis (CH_3CN); λ_{max} (ε_M, $M^{-1}cm^{-1}$): 289 (17000), 347 (5000), 559 (8000), 658 (7100). LTQ-MS (m/z): positive ion mode: {*n*-$Bu_3NH\}^+$, 186.25 (base); {(*n*-$Bu_3NH)_2[V(C_6H_4O_2)_3]\}^+$, 748.46; negative ion mode: $\{H[V(C_6H_4O_2)_3]\}^-$, 376.08. Combustion analyses calculated for $C_{42}H_{56}Br_{12}N_2O_6V$ (found): 29.77 (29.94) % C; 3.33 (3.17) % H; 1.65 (1.46) % N.

Determination of Density of d[14]-*o*-terphenyl. 1 g d[14]-*o*-terphenyl was placed in a 20-mL scintillation vial and the mass was recorded. Next, the vial was heated to 60 °C for d[14]-*o*-terphenyl to melt. 0.1 mL d[14]-*o*-terphenyl was removed via a micropipette. The mass difference was recorded and the density of d[14]-*o*-terphenyl could be calculated. This process was repeated two more times to yield an average density of 1.29(5) g/cm^3.

X-ray Data Collection, Structure Solution and Refinement for 2, 4, and 5. The diffraction data were collected at the X-Ray Diffraction facility of the Central Instrument Facility at Colorado State University. Data for **2, 4,** and **5** were collected on a Bruker D8 Quest ECO single-crystal X-ray diffractometer equipped with Mo Kα (λ = 0.71073 Å). Data were collected and integrated using Bruker Apex 3 software. Absorption correction were applied using SADABS.[6] Space group assignments were determined by examination of systematic absences, E-statistics, and successive refinement of the structures. Crystal structures were solved using SHELXT and refined with the aid of successive difference Fourier maps by SHELXL operated in conjunction with OLEX2 software.[7–9] None of the crystals demonstrated decay by X-ray radiation over the course of the experiment. Hydrogen atoms were placed in ideal positions and refined using a riding model for all structures. In **2**, two of the alkyl chains were each disordered over two positions and were modeled using free variables. In one of the alkyl chains the disordered carbons freely modeling the two positions yielded occupancies of 0.393(14) and 0.607(14), respectively. The methyl group on the end of the other alkyl chain yielded occupancies of 0.504(14) and 0.496(14), respectively. One of the 4,5-dibromocatecholate ligands in **2** was disordered over two positions by rotational symmetry and was modeled using free variables yielding occupancies 0.65(5), 0.64(4), 0.56(3), 0.55(4), and 0.528(3) for C1, C1A, C2A, C2B, and Br, respectively. In **4**, two of the alkyl chains

on the counteraction was disordered over two positions and were modeled using free variables yielding occupancies of 0.69(3) and 0.31(3). In **5** one of the alkyl chains on the counteraction was disordered over two positions and were modeled using free variables yielding occupancies of 0.66(3) and 0.34(3). See Tables S1–S4 for refinement details. Crystallographic information files for **2**, **4**, and **5** are available in the CSD at accession numbers 1921675-1921677.

Electron Paramagnetic Resonance. EPR spectra collected herein were simulated using Easyspin[10] with the function pepper (frozen solution and solid) and were refined using the function esfit or simulations of the experimental data. All samples were prepared under an inert atmosphere. Samples **1**, **2**, **3**, and **5** for pulsed EPR studies (1 mM) were prepared by first loading 20 μL 1 mM THF solution of **1** by a micropipette into a 4 mm OD quartz EPR tube. Following removal of THF under reduced pressure, 0.0232 g *o*-terphenyl or 0.0258 g d^{14}-*o*-terphenyl (20 μL when molten) was loaded into the same tube. Sample **4** is less soluble in non-polar solvents, so the aforementioned preparation was unsuccessful. Sample **4** for pulsed EPR studies was prepared by first loading 0.0258 g d^{14}-*o*-terphenyl (20 μL when molten), then adding a 1 mM solution of **4** in THF to the same tube and them removing THF under reduced pressure. These tubes were flame sealed under dynamic vacuum (< 50 mTorr) and placed in a 65−70 °C oil bath until a clear dark blue solution formed. While sample tubes prepared in this manner can be stored at room temperature for an extended period without compound decomposition, the quality of glass appeared to decay with extended time on the basis of spectral quality. Hence, prior to each measurement, the samples were remelted and frozen.

All pulsed EPR data were collected at the National High Magnetic Field Laboratory[11] (NHMFL, Tallahassee, FL, USA) on a custom built 120/240/336 GHz EPR spectrometer.[12,13]

Sample tubes were gently melted using a heat gun to afford a homogeneous dark blue solution, then quickly inserted into the resonator to ensure glassing behavior. The data collected were processed using an EPR measurement program locally developed at NHMFL, Matlab 2018b, and Origin Pro 2018b software packages.[14,15]

T_1 data were collected on the most intense resonance of the EDFS spectra at 4.4 T at 120 GHz, via an inversion recovery sequence ($\pi - T - \pi/2 - \tau - \pi - \tau -$ echo). The length of the three pulses, $\pi - \pi/2 - \pi$, are 900− 600 − 900 ns with a starting T value of 10300 ns and τ of 700 ns. The inversion recovery data were fit accounting for spectral diffusion with the following equation:

$$\mathrm{I}(t) = -A\left[e^{-\left(\frac{t}{T_1}+\sqrt{\frac{t}{q}}\right)} - \mathrm{I}(0) - 1\right]$$

The temperature-dependent T_1 data were fit using the following equation performed in Matlab software:

$$\frac{1}{T_1} = A_{\mathrm{dir}}T + A_{\mathrm{Ram}}\left(\frac{T}{\theta_{\mathrm{D}}}\right)^9 J_8\left(\frac{\theta_{\mathrm{D}}}{T}\right)$$

Here, T is temperature, A_{dir} is the direct process coefficient, A_{Ram} is the Raman process coefficient, θ_{D} is the Debye temperature, and $J_8\left(\frac{\theta_{\mathrm{D}}}{\mathrm{T}}\right)$ is the transport integral:

$$J_8\left(\frac{\theta_{\mathrm{D}}}{T}\right) = \int_0^{\theta_{\mathrm{D}}/T} x^8 \frac{\mathrm{e}^x}{(\mathrm{e}^x - 1)^2}\,\mathrm{d}x$$

Variable temperature T_1 data presented challenges in fitting. Due to the enhanced relaxation rate of spin-lattice relaxation with 120 GHz frequencies, measurements were restricted to below 40 K. Furthermore, **4** has a much shorter T_m than the rest of the series, precluding higher temperature measurements of T_1 at 120 GHz. We successfully fit the data using the Raman and direct processes, using Raman parameters (A_{Ram} and θ_{D} to 1.8×10^6 (s^{-1}) and 95 K, respectively) close to those obtained from the X-band analyses previously reported

by some of us and others.[16] Since the Raman process is *field-independent,* this method was done to see how the A_{dir} value changed. By restricting A_{Ram} and θ_D to the X-band values, we obtain an A_{dir} value of 197(82) $K^{-1}s^{-1}$. When A_{Ram} and θ_D values were unrestricted, we obtained equivalent values within the error of the parameters (see Table S6).

The discrepancy between the direct process coefficients (A_{dir}) between this study and the X-band analyses of $(Bu_3NH)_2[V(C_6H_4O_2)_3]$ (**1**) deserves additional comment. The direct process field dependence is B^2, hence:

$$\frac{1}{T_1} = A_{\mathrm{dir}} B^2 T$$

On account of this equation, one might expect that a fit of the T_1 temperature-dependent data would yield the same A_{dir} between the X-band and 120 GHz data of **1** following a switch of B, considering that the measurements are of samples of the same concentration in the same frozen matrix. The values of A_{dir}, between X-band data and the present data are close, but not the same, from this approach (27(2) $K^{-1}s^{-1}T^{-2}$ for the X-band data and 10(4) $K^{-1}s^{-1}T^{-2}$ for the 120 GHz data). Note that the original reported 3.2(2) $K^{-1}s^{-1}$ value of A_{dir} in ref. 16 does not account for the field strength. Additional analyses are required to understand why this change in A_{dir} is observed between the two fields for the same complex. However, we note that the nature of the probed resonances at 120 GHz and X-band change substantially, because the transitions are heavily overlapped at high-field versus low-field (Fig. S18). Such overlap might (1) change the A_{dir} parameter or (2) enable the operation of other relaxation processes. In this latter case, the effect of such unmodeled processes on the fit could be manifest in a change to A_{dir}. A clear solution to this problem is the ability to collect far more detailed T_1 data sets – but extremely low signal-to-noise and limited instrument time deem this experimental design unfeasible for now.

T_m data were collected on the most intense resonance (4.4 T at 120 GHz) in the EDFS spectra via a Hahn echo sequence ($\pi/2 - \tau - \pi - \tau -$ echo) with a 4-step phase cycle with microwave pulses of 600 ($\pi/2$) and 900 ns (π) and an interpulse time (τ) of 700 ns. The Hahn echo decay data were fit using the stretched exponential equation:

$$\mathrm{I}(\tau) = I(0) - Ae^{-\left(\frac{2\tau}{T_m}\right)^{\beta}}$$

Here, β is the stretch parameter. The low temperature data were better fit with stretched exponential equation due to the dominant nuclear spin diffusion. We found that β approached 1 as the temperature increased, indicating that a conventional single exponential fit could be used. Nevertheless, we used the stretched exponential fit throughout the whole temperature range for consistency.

Owing to the long pulse lengths and 100 ns deadtime, T_m values below 200-300 ns were in general extremely difficult to observe, requiring prohibitively long data acquisition times. This factor limited the temperature range for a usable echo to only the lowest temperatures, hence all analyses generally stopped by 40 K, lower for **4**, which displayed much shorter T_m values than the rest of the series.

Other Physical Measurements. ^{1}H NMR spectra were collected on a Bruker Avance NEO 400 MHz spectrometer. The spectra were referenced using residual protiated solvent signal as an internal standard ($CDCl_3$, 7.26 ppm). Combustion analyses were performed by Midwest Microlab (Indianapolis, IN, USA). Infrared spectra were recorded on a Bruker TENSOR II FTIR spectrometer. Electronic absorption spectra were recorded on acetonitrile solutions of **1**−**5** with a Hewlett-Packard 8453 spectrophotometer using air-free quartz cuvettes with a 1 cm path length. Linear trap quadruple mass spectrometry (LTQ-MS) measurements were performed on acetonitrile

solutions of **1**−**5** with a Thermo-Finnigan LTQ LC/MS-MS at the Central Instrument Facility (CIF) of the Colorado State University.

Table S1. Crystallographic information for the structural refinement of **2**.

Empirical formula	$H_{64}C_{42}N_2Br_3O_6V$
Formula weight	983.62
Temperature	116(2)
Crystal system	tetragonal
Space group	$P4_32_12$
a	14.6090(13) Å
b	14.6090(13) Å
c	21.144(2) Å
α	90°
β	90°
γ	90°
Volume	4512.6(9) $Å^3$
Z	4
ρ_{calc}	1.448 g/cm^3
μ	2.921 mm^{-1}
F(000)	2024
Crystal color	Dark blue
Crystal size	0.185 × 0.162 × 0.159 mm^3
Radiation	MoKα (λ = 0.71073 Å)
2θ range for data collection	3.388 to 60.068°
Index ranges	–19 ≤ h ≤ 19, –14 ≤ k ≤ 19, –28 ≤ l ≤ 20
Reflections collected	16717
Independent collections	5621 [R_{int} = 0.0274, R_{sigma} = 0.0546]
Data/restraints/parameters	5621/2/276
Goodness-of-fit on F^2	1.131
Final R indexes [I ≥ 2σ (I)]	R_1 = 0.0376, wR_2 = 0.0955
Final R indexes [all data]	R_1 = 0.0566, wR_2 = 0.1261
Largest diff. peak/hole	0.86/–0.61 e $Å^{-3}$
Flack parameter	0.000(4)

Table S2. Crystallographic information for the structural refinement of **4**.

Empirical formula	$H_{62}C_{42}N_2Br_6O_6V$
Formula weight	1221.33
Temperature	120(2)
Crystal system	tetragonal
Space group	$P4_32_12$
a	10.7250(6) Å
b	10.7250(6) Å
c	42.298(3) Å
α	90°
β	90°
γ	90°
Volume	4865.3(6) $Å^3$
Z	4
ρ_{calc}	1.667 g/cm^3
μ	5.176 mm^{-1}
F(000)	2436
Crystal color	Dark blue
Crystal size	0.156 × 0.154 × 0.146 mm^3
Radiation	MoKα (λ = 0.71073 Å)
2θ range for data collection	3.852 to 51.358°
Index ranges	$-13 \leq h \leq 13, -13 \leq k \leq 13, -51 \leq l \leq 51$
Reflections collected	408403
Independent collections	4625 [R_{int} = 0.0872, R_{sigma} = 0.0223]
Data/restraints/parameters	4625/2/261
Goodness-of-fit on F^2	1.148
Final R indexes [I ≥ 2σ (I)]	R_1 = 0.0508, wR_2 = 0.1459
Final R indexes [all data]	R_1 = 0.0522, wR_2 = 0.1500
Largest diff. peak/hole	0.95/–0.69 e $Å^{-3}$
Flack parameter	0.059(5)

Table S3. Crystallographic information for the structural refinement of **5**.

Empirical formula	$H_{56}C_{42}N_2Br_{12}O_6V$
Formula weight	1694.63
Temperature	145 (2)
Crystal system	triclinic
Space group	*P*–1
a	12.039(5) Å
b	12.432(6) Å
c	37.11(2) Å
α	83.93(3)°
β	89.04(2)°
γ	84.219(19)°
Volume	5495(5) Å^3
Z	4
ρ_{calc}	2.048 g/cm^3
μ	8.950 mm^{-1}
F(000)	51
Crystal color	Dark blue
Crystal size	0.372 × 0.235 × 0.142 mm^3
Radiation	MoKα (λ = 0.71076 Å)
2θ range for data collection	7.566 to 52.988°
Index ranges	$-15 \leq h \leq 15, -15 \leq k \leq 15, -46 \leq l \leq 46$
Reflections collected	46089
Independent collections	21824 [R_{int} = 0.0494, R_{sigma} = 0.0757]
Data/restraints/parameters	21824/0/1148
Goodness-of-fit on F^2	1.154
Final R indexes [$I \geq 2\sigma$ (I)]	R_1 = 0.0653, wR_2 = 0.1662
Final R indexes [all data]	R_1 = 0.0829, wR_2 = 0.1685
Largest diff. peak/hole	1.24/–1.54 e Å^{-3}
Flack parameter	0.060(5)

Table S4. Continuous-shape-measurement (CSM) (SHAPE 2.0 software) analysis results for **1**, **2**, **4** and **5**.

Complex	SHAPE analysis octahedral	SHAPE analysis trigonal prismatic
**1**	1.67	8.83
**2**	2.39	7.64
**4**	3.97	5.45
**5**	1.41	10.52

Table S5. EDFS spin Hamiltonian parameters for **1**–**5** in d[14]-*o*-terphenyl collected at 5 K. The standard errors for these parameters were not available from the simulating process.

Complex	**1**	**2**	**3**	**4**	**5**
g_x	1.939	1.938	1.938	1.943	1.934
g_y	1.923	1.920	1.921	1.924	1.914
g_z	1.990	1.990	1.990	1.991	1.989
g_x strain	0.0042	0.0045	0.0045	0.0052	0.0042
g_y strain	0.0129	0.028	0.028	0.020	0.0190
g_z strain	0	0	0	0	0
A_x (MHz)	301	280	280	285	285
A_y (MHz)	348	365	365	375	355
A_z (MHz)	53	60	60	50	70

*Changes in *A*-strain resulted in no change in the simulated spectrum.

Table S6. Fit T_1 values from the fitting function: $I(t) = -A\left[e^{-\left(\frac{t}{T_1}+\sqrt{\frac{t}{q}}\right)} - I(0) - 1\right]$. The standard error for each fit is reported in parentheses.

1 in d^{14}-*o*-terphenyl:

T (K)	T_1 (ms)	q	T(K)	T_1 (ms)	q
4	1.23(6)	7(1)	15	0.26(1)	0.8(1)
5	1.15(3)	5.6(6)	20	0.09(1)	14(40)
10	0.43(1)	3.8(5)			

2 in d^{14}-*o*-terphenyl:

T (K)	T_1 (ms)	q	T(K)	T_1 (ms)	q
5	1.05(2)	7.0(6)	25	0.076(4)	3(3)
10	0.43(1)	3.0(3)	30	0.055(4)	5(9)
15	0.254(9)	1.0(1)	40	0.025(4)	15(172)
20	0.139(7)	2.0(9)			

3 in d^{14}-*o*-terphenyl:

T (K)	T_1 (ms)	q	T(K)	T_1 (ms)	q
4	2.55(9)	15(2)	15	0.24(2)	4(2)
5	2.3(1)	9(1)	20	0.15(2)	3(3)
10	0.50(2)	2.4(4)	30	0.066(3)	3(1)

4 in d^{14}-*o*-terphenyl:

T (K)	T_1 (ms)	q	T(K)	T_1 (ms)	q
5	1.91(8)	3.9(4)	15	0.41(16)	0.9(3)
10	1.22(7)	1.4(1)			

5 in d^{14}-*o*-terphenyl:

T (K)	T_1 (ms)	q	T(K)	T_1 (ms)	q
5	1.20(3)	3.2(4)	20	0.12(3)	0.5(8)
10	0.456(8)	2.5(2)	30	0.03(1)	0.2(2)
15	0.256(9)	0.1(3)			

Table S7. Fit parameters for the temperature dependence of T_1; see "EPR measurements" section for fitting equation. The standard error for each fit is reported in parentheses.

A_{dir} (K^{-1} s^{-1} T^{-2})	197(82)
A_{Ram} (s^{-1})	1.8(1)×10^6
θ_D (K)	95(5)

Table S8. Fit T_m values (in μs) and the stretch parameters (β) from the stretched exponential fitting function: $I(2\tau) = I(0) - Ae^{-(\frac{2\tau}{T_m})^{\beta}}$. The standard error for each fit is reported in parentheses.

1 in *d*[14]-*o*-terphenyl:

T (K)	T_m (μs)	β	T(K)	T_m (μs)	β
4	5.44(2)	0.660(2)	15	2.81(4)	0.80(1)
5	4.82(3)	0.678(3)	20	2.45(4)	0.86(2)
6	4.56(2)	0.707(3)	25	2.14(4)	0.91(2)
8	3.92(3)	0.740(4)	30	1.86(3)	0.94(2)
10	3.39(3)	0.769(6)			

2 in *d*[14]-*o*-terphenyl:

T (K)	T_m (μs)	β	T(K)	T_m (μs)	β
4	5.48(8)	0.659(7)	20	2.51(4)	0.81(1)
5	5.36(9)	0.70(1)	30	2.02(5)	0.87(3)
6	5.03(7)	0.708(7)	40	1.69(6)	0.89(5)
8	4.13(6)	0.737(9)	50	1.34(9)	0.82(7)
10	3.63(5)	0.78(1)	60	1.3(1)	1.1(2)
15	2.93(4)	0.82(1)			

3 in *d*[14]-*o*-terphenyl:

T (K)	T_m (μs)	β	T(K)	T_m (μs)	β
4	4.57(8)	0.629(7)	20	2.66(7)	0.78(2)
5	4.36(8)	0.646(8)	25	2.47(9)	0.79(3)
6	4.42(7)	0.75(1)	30	2.27(5)	0.85(2)
8	3.90(7)	0.69(1)	40	1.96(4)	0.86(2)
10	3.61(6)	0.72(1)	50	1.58(5)	0.90(3)
15	3.00(6)	0.76(1)	60	1.47(9)	0.96(7)

4 in *d*[14]-*o*-terphenyl:

T (K)	T_m (μs)	β	T(K)	T_m (μs)	β
4	2.97(3)	1.05(2)	10	1.63(7)	1.12(6)
5	2.75(4)	1.03(2)	15	0.99(9)	1.1(1)

5 in *d*[14]-*o*-terphenyl:

T (K)	T_m (μs)	β	T(K)	T_m (μs)	β
4	4.86(4)	0.591(3)	15	2.96(2)	0.802(5)
4.5	5.27(3)	0.666(2)	20	2.44(2)	0.833(8)
5	5.10(4)	0.651(3)	30	1.90(4)	0.87(2)
6	4.54(3)	0.696(3)	40	1.55(7)	0.97(5)
8	4.19(2)	0.729(3)	50	1.26(6)	1.03(6)
10	3.71(2)	0.750(3)			

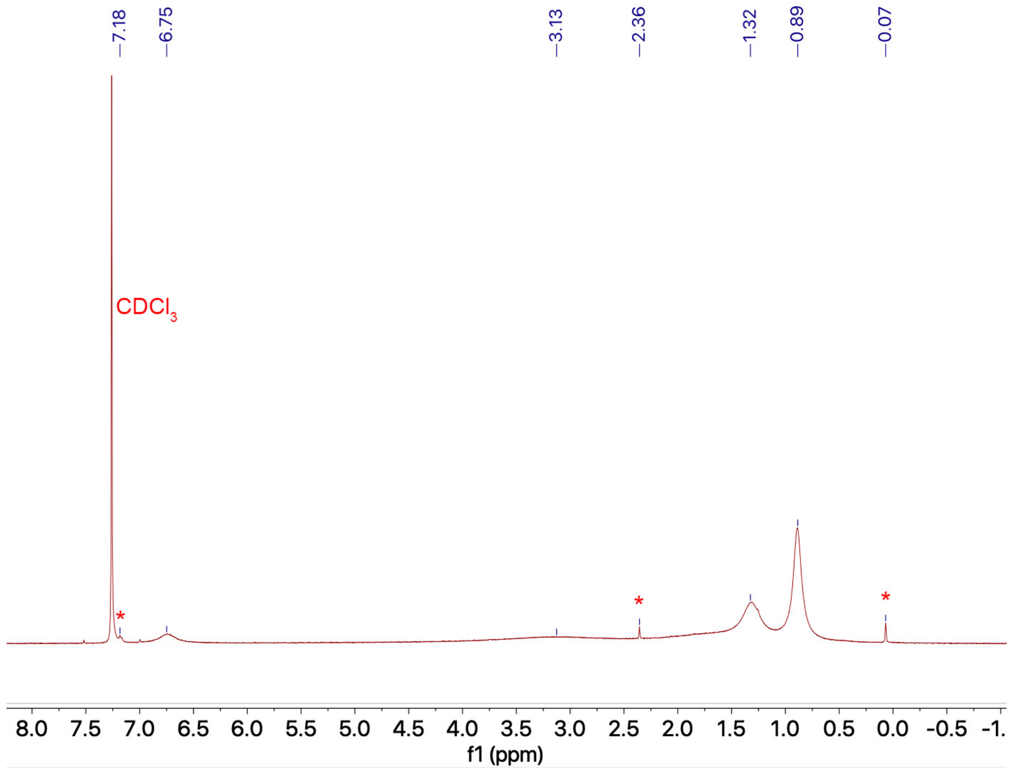

Fig. S1. ^{1}H NMR spectrum of **2** in $CDCl_3$. No signal was observed outside this spectral range. Asterisks denote impurities in the NMR solvent.

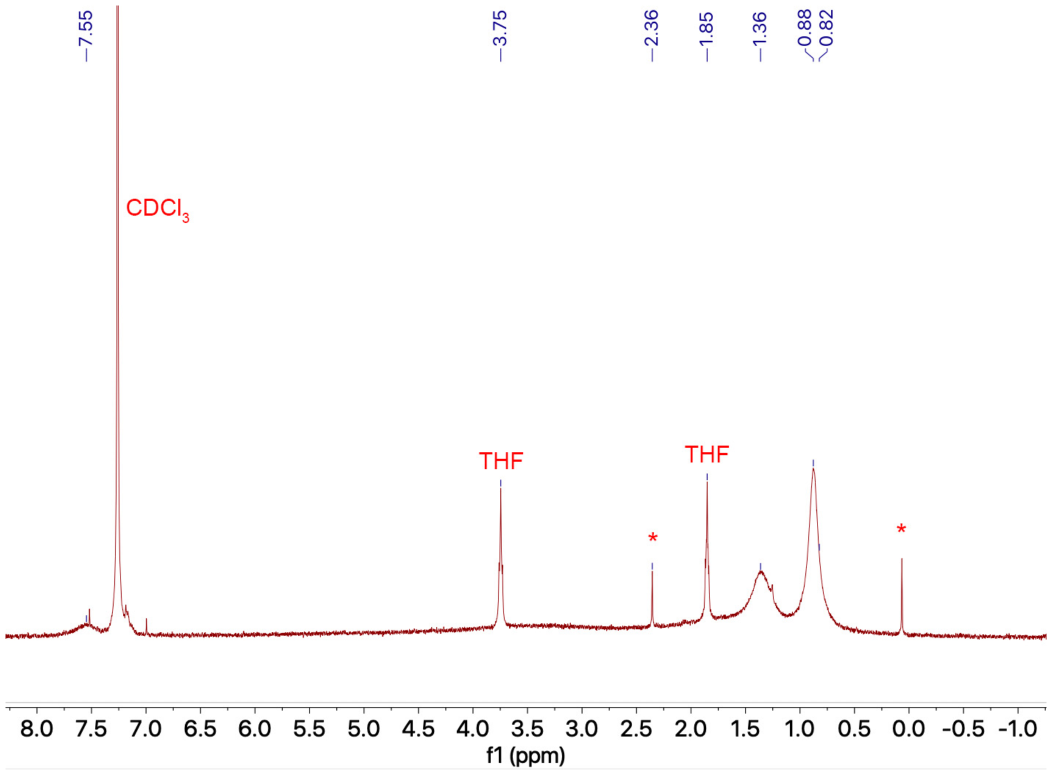

Fig. S2. ^{1}H NMR spectrum of **3** in $CDCl_3$. No signal was observed outside this spectral range. Asterisks denote impurities in the NMR solvent.

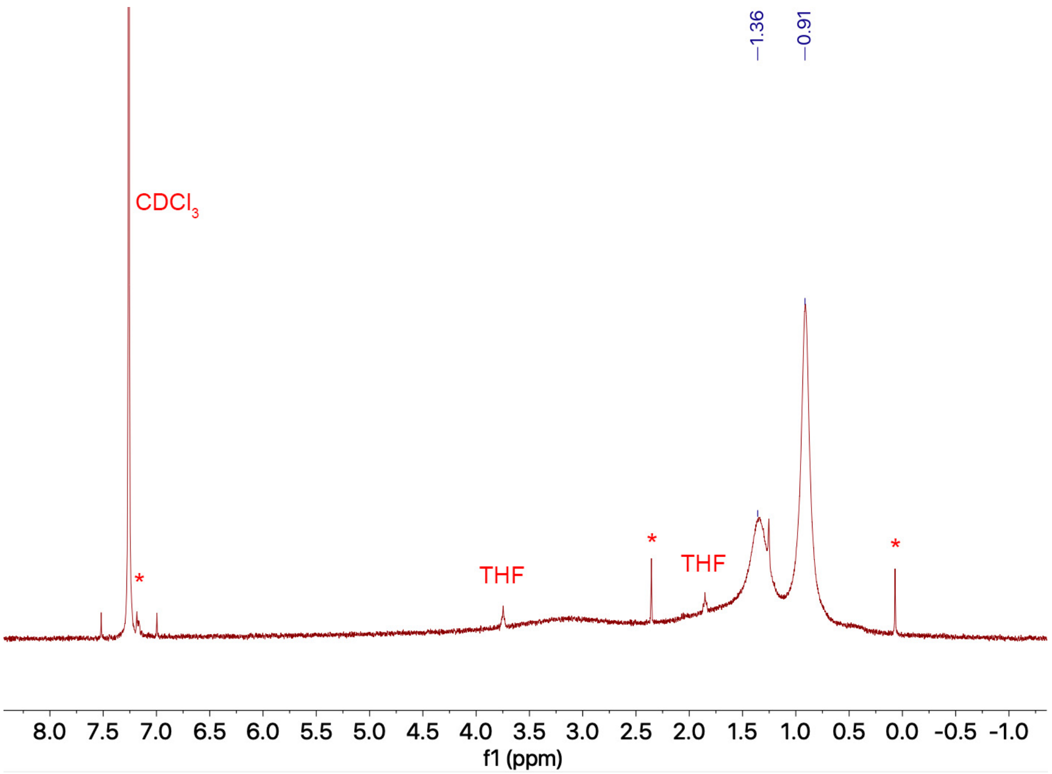

Fig. S3. ^{1}H NMR spectrum of **4** in $CDCl_3$. No signal was observed outside this spectral range. Asterisks denote impurities in the NMR solvent.

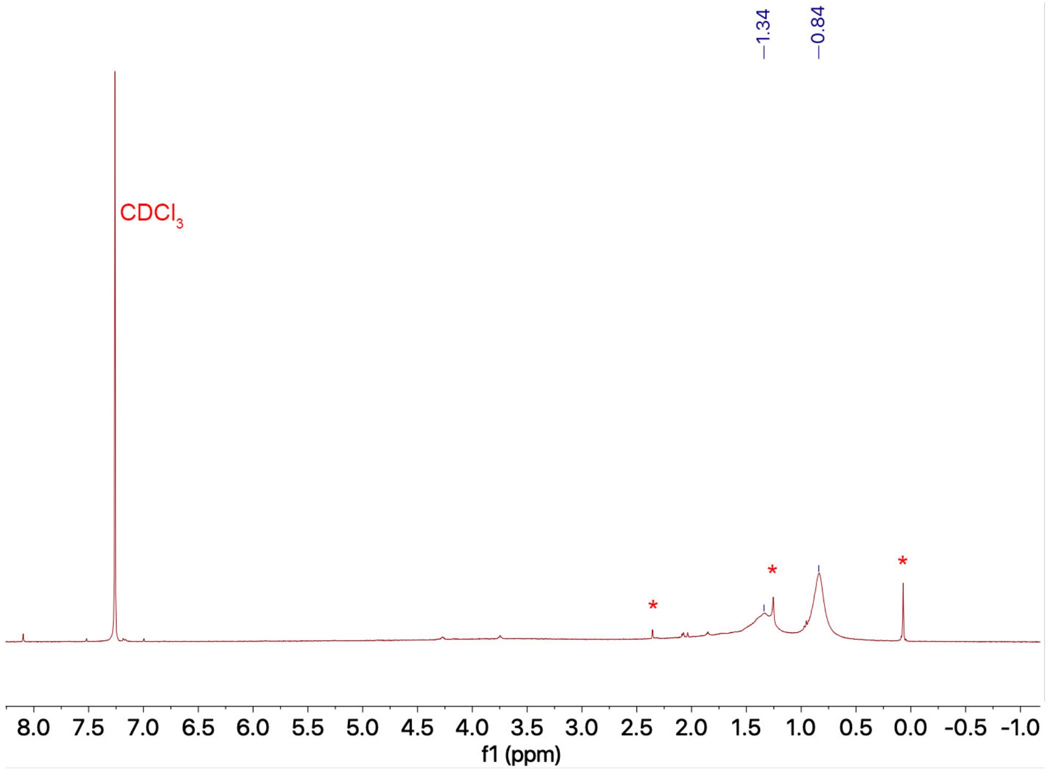

Fig. S4. ^{1}H NMR spectrum of **5** in $CDCl_3$. No signal was observed outside this spectral range. Asterisks denote impurities in the NMR solvent.

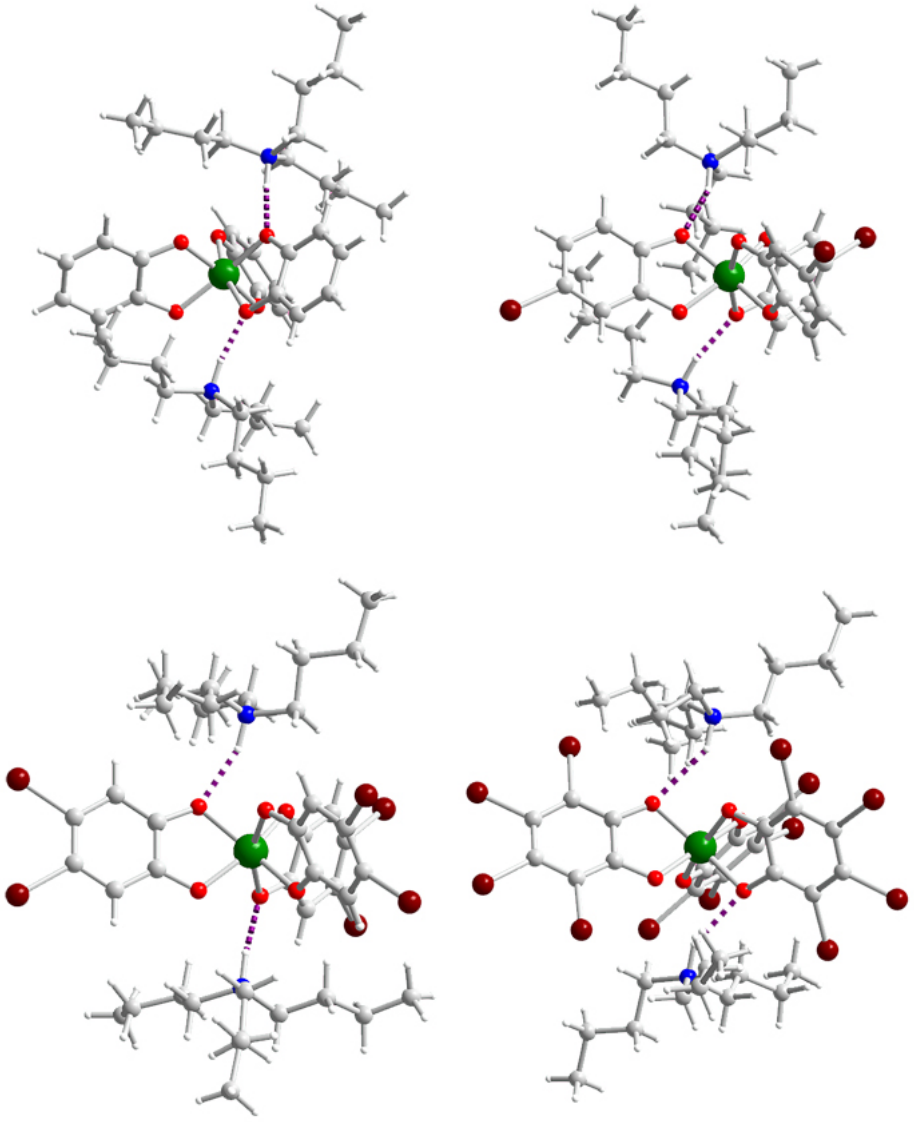

Figure S5. Depiction of hydrogen bonding interactions in **1**, **2**, **4**, and **5**. Molecular structures of the V(IV) complexes in **1** (top left) (ref. 16), **2** (top right), **4** (bottom left), and **5** (bottom right), as determined from the crystal structures of these compounds. Green, maroon, red, gray, and white spheres represent vanadium, bromine, oxygen, carbon, and hydrogen atoms, respectively. Dashed lines represent the hydrogen bonding interactions.

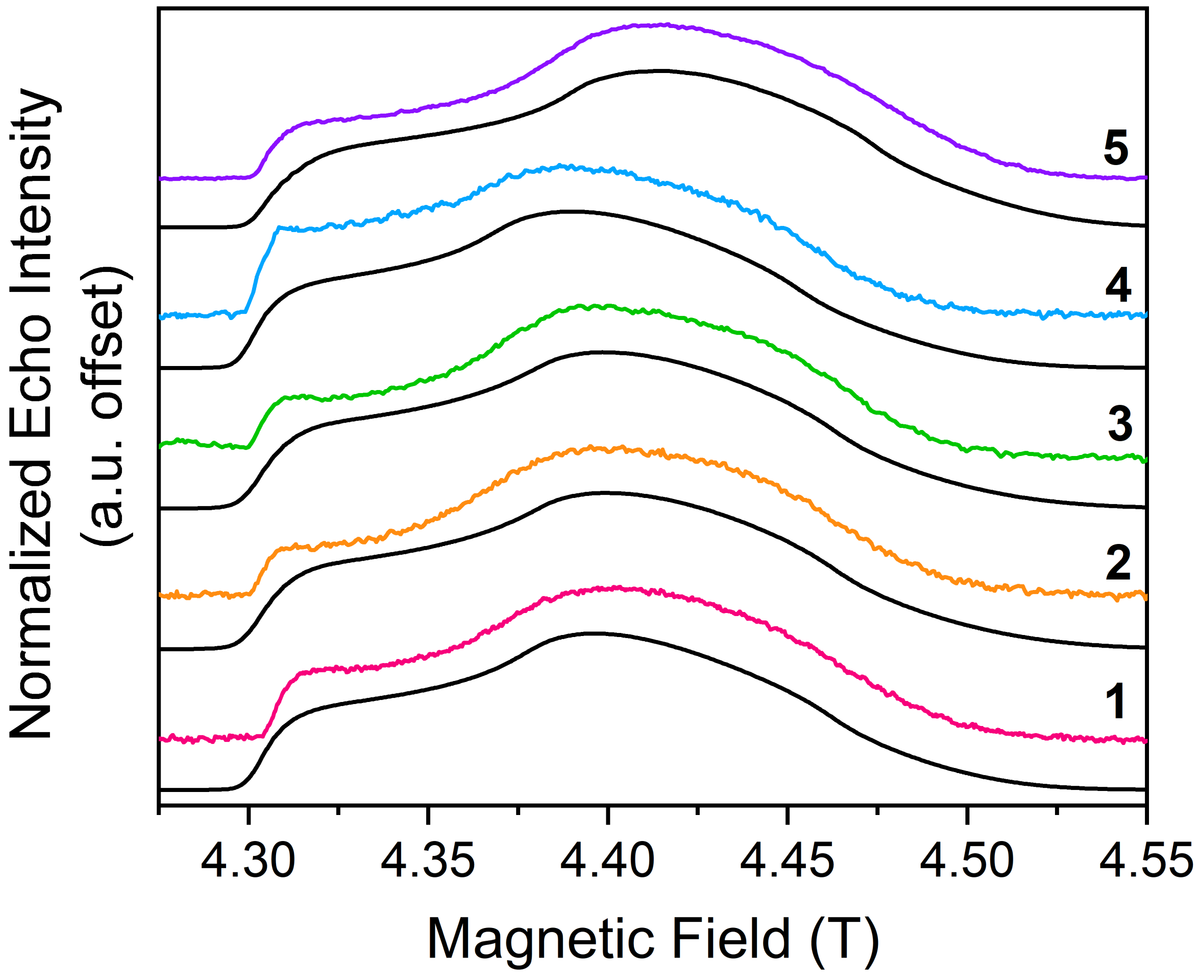

Fig. S6. Echo-detected, field-swept spectra at 120 GHz of **1**–**5** in d[14]–*o*–terphenyl solutions (color) at 5 K and simulations (black). Simulated spin Hamiltonian parameters are given in Table S6.

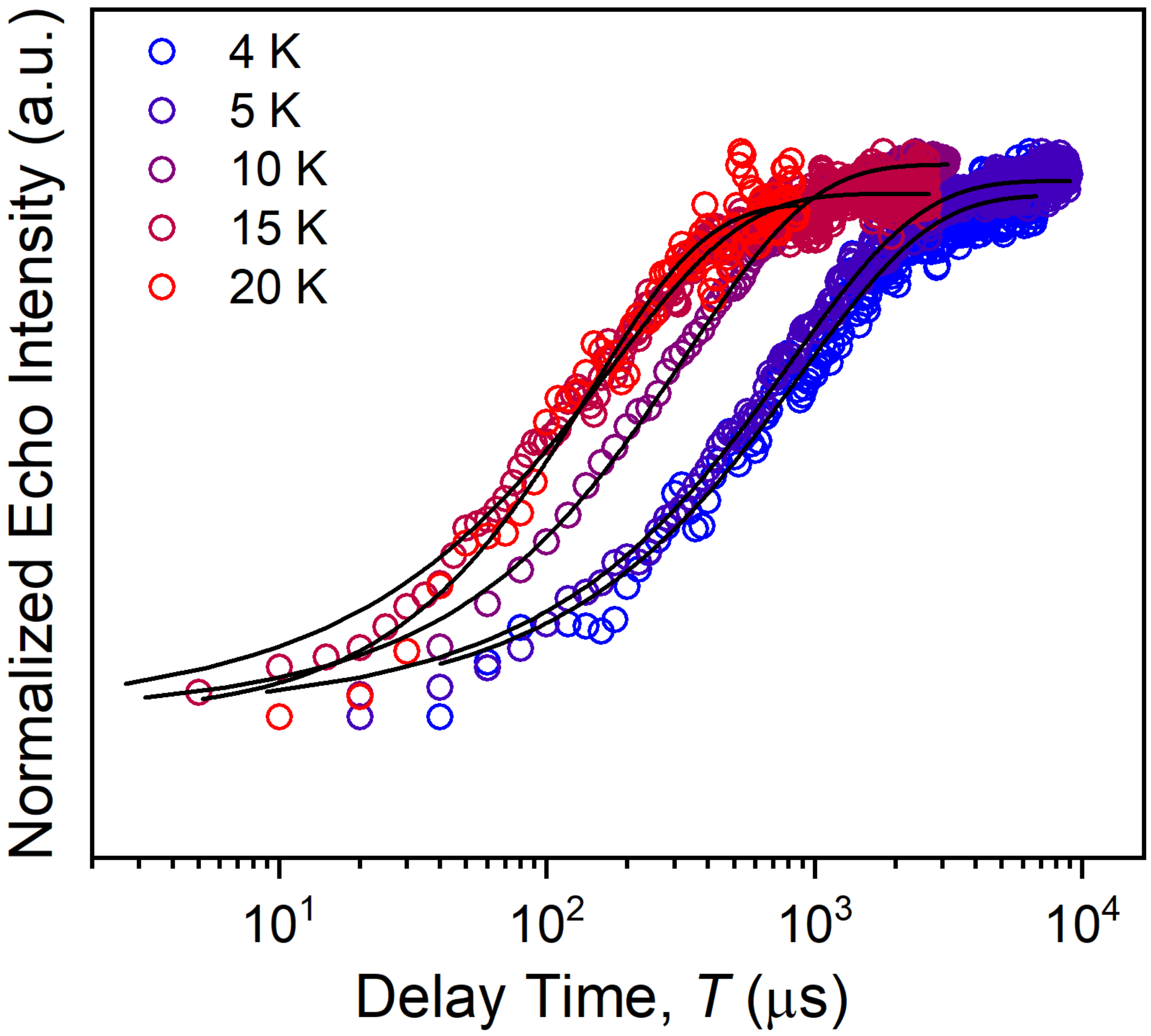

Figure S7. Selected variable temperature inversion recovery curves (color traces) and fits (black traces) for **1** in d[14]-*o*-terphenyl.

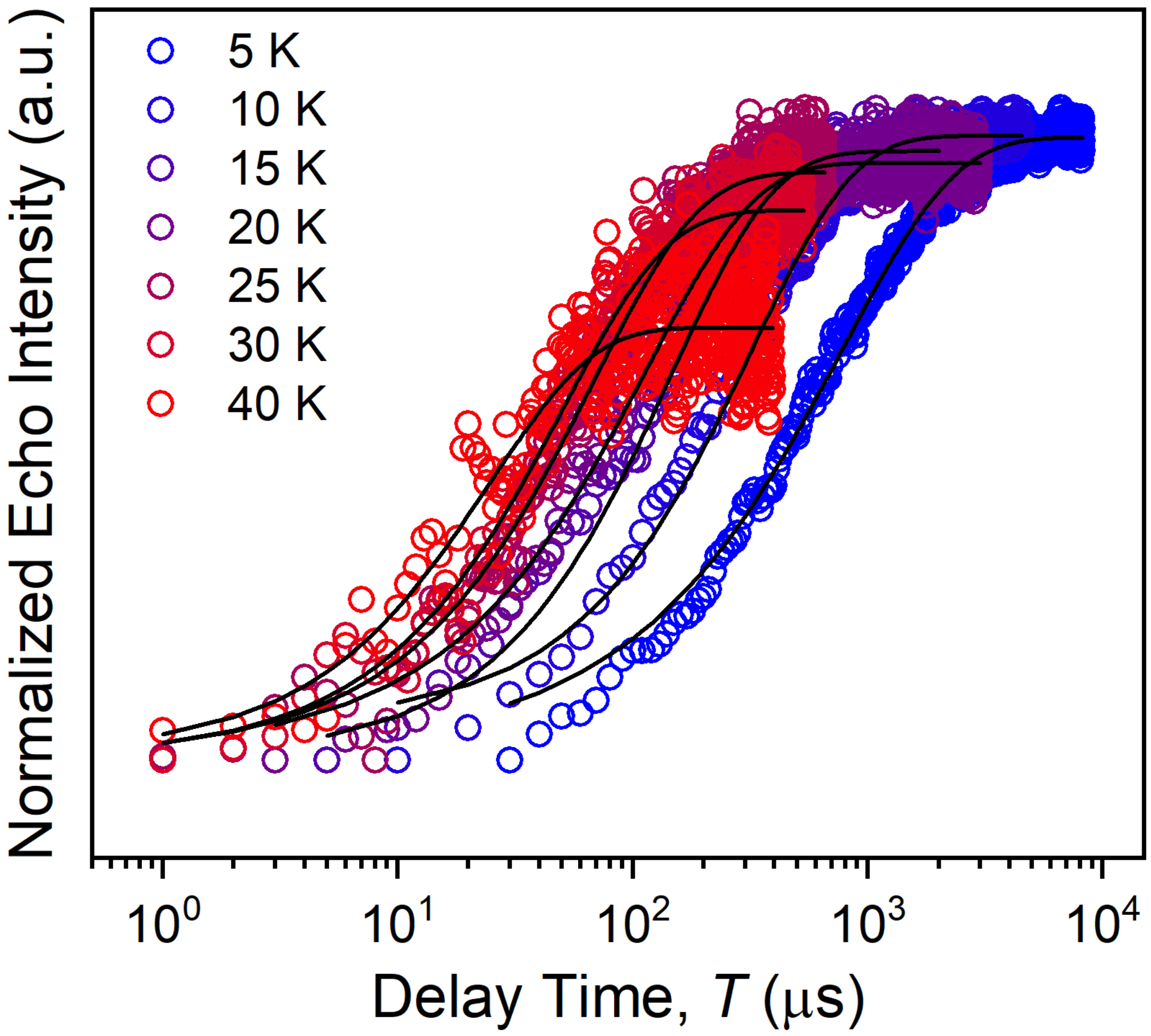

Figure S8. Selected variable temperature inversion recovery curves (color traces) and fits (black traces) for **2** in d^{14}-*o*-terphenyl.

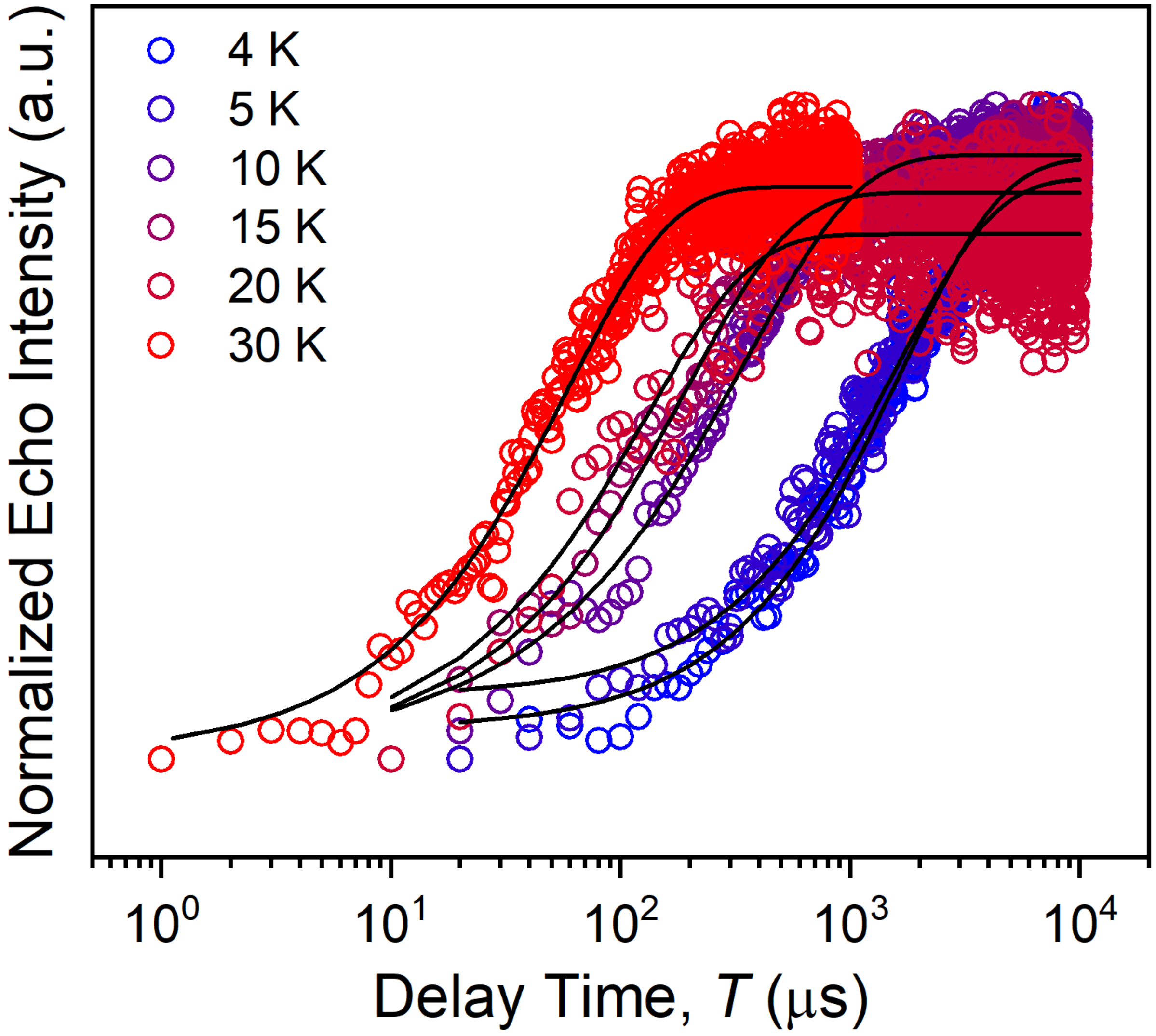

Figure S9. Selected variable temperature inversion recovery curves (color traces) and fits (black traces) for **3** in d[14]-*o*-terphenyl.

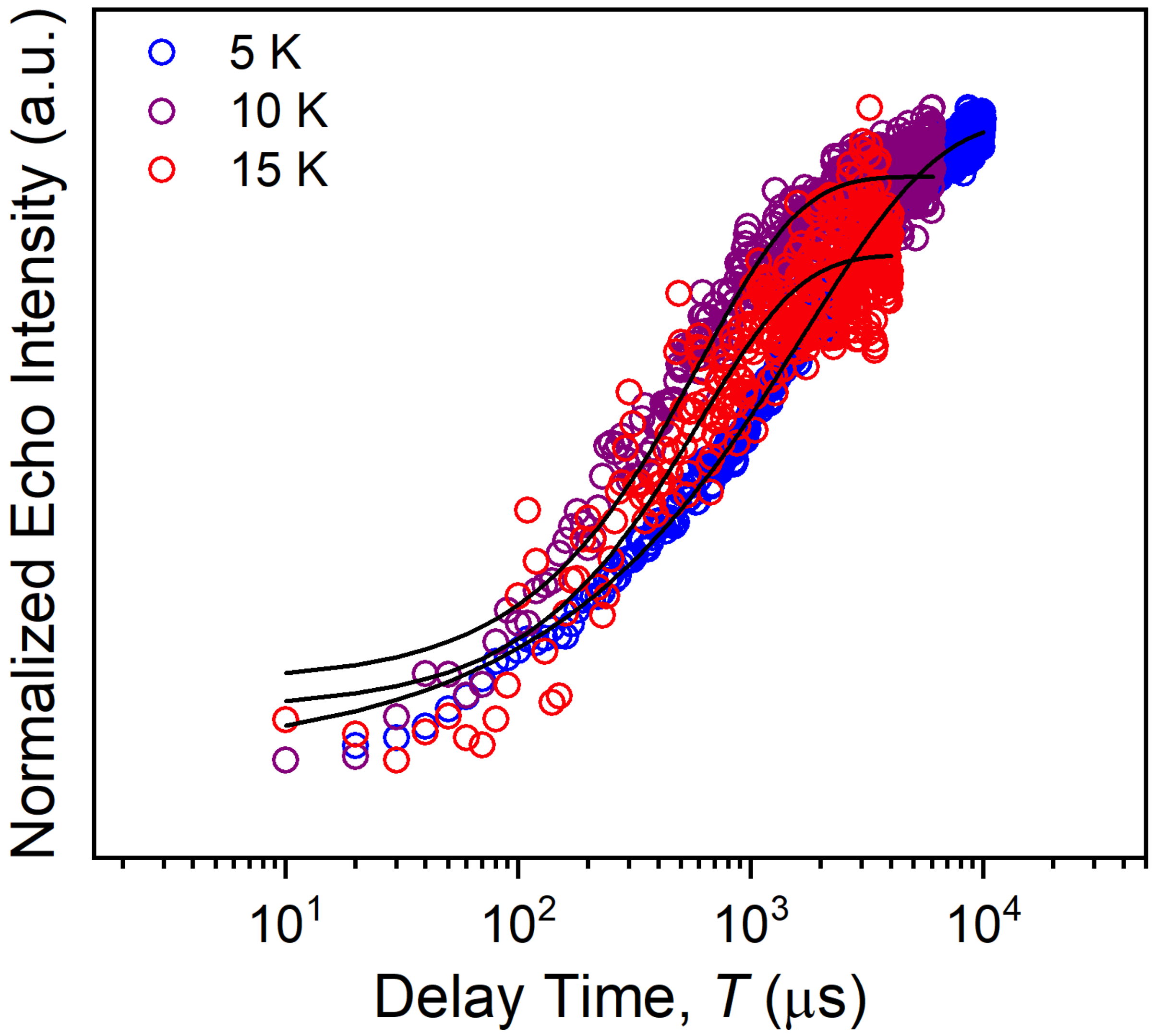

Figure S10. Selected variable temperature inversion recovery curves (color traces) and fits (black traces) for **4** in d^{14}-*o*-terphenyl.

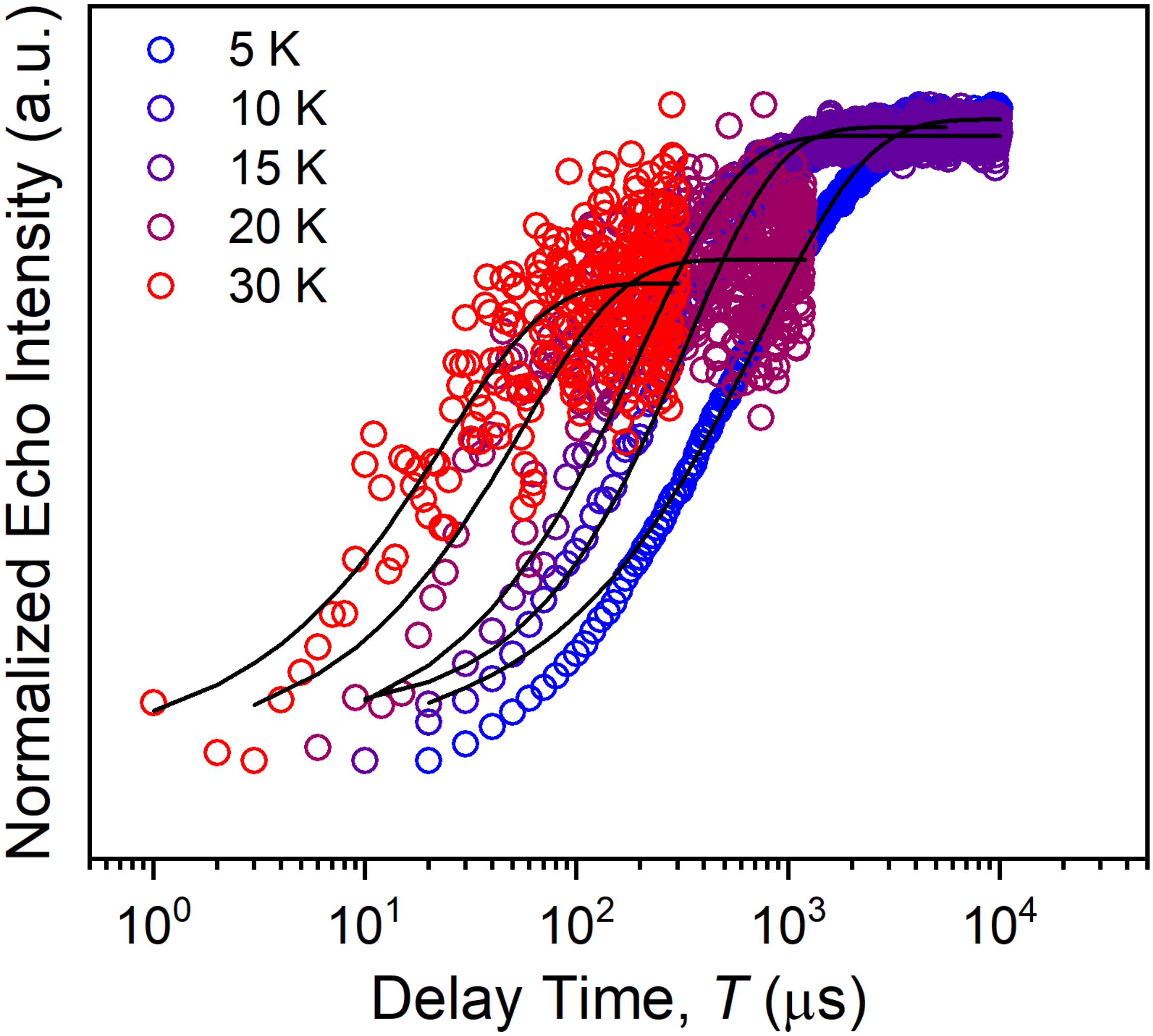

Figure S11. Selected variable temperature inversion recovery curves (color traces) and fits (black traces) for **5**.

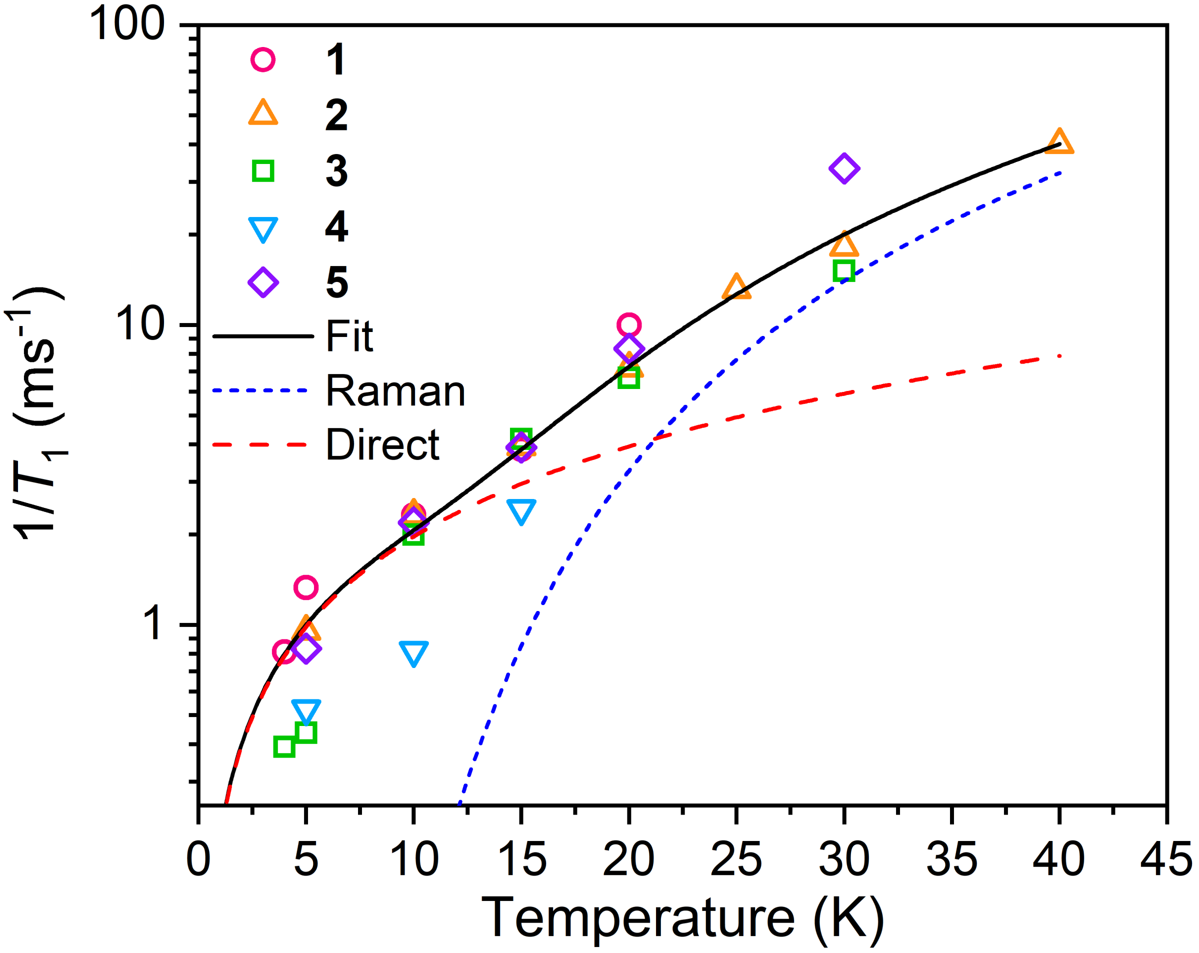

Figure S12. Variable temperature T_1 data showing the individual relaxation processes for **1**–**5**. Black line represents the best fit to the data as a sum of the direct and Raman processes. The individual components are shown in dashed lines. The equation for modelling the temperature dependent T_1 data is in the Electron Paramagnetic Resonance section of the ESI with the fit parameters in Table S6.

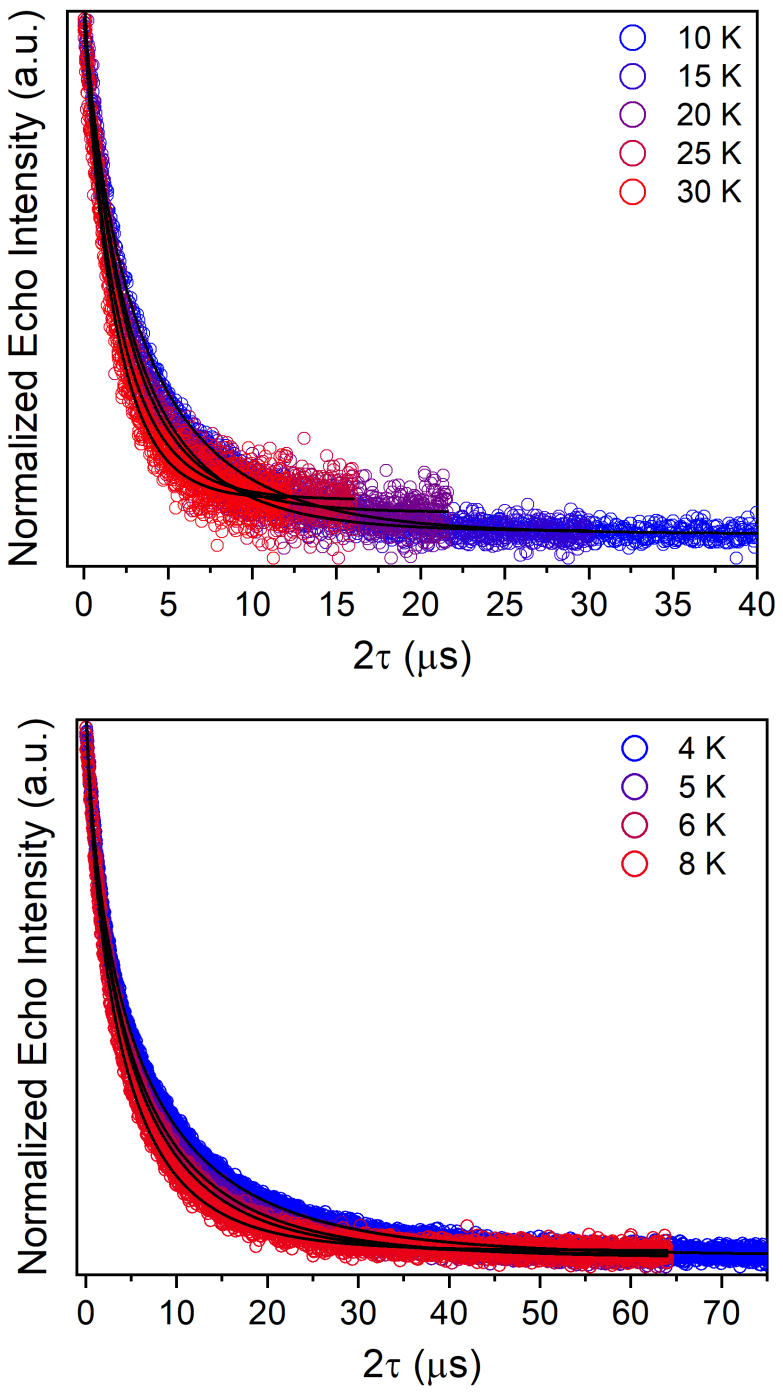

Fig. S13. Selected variable temperature Hahn echo decay curve (color traces) and fits (black traces) for **1** in d^{14}-*o*-terphenyl at 120 GHz.

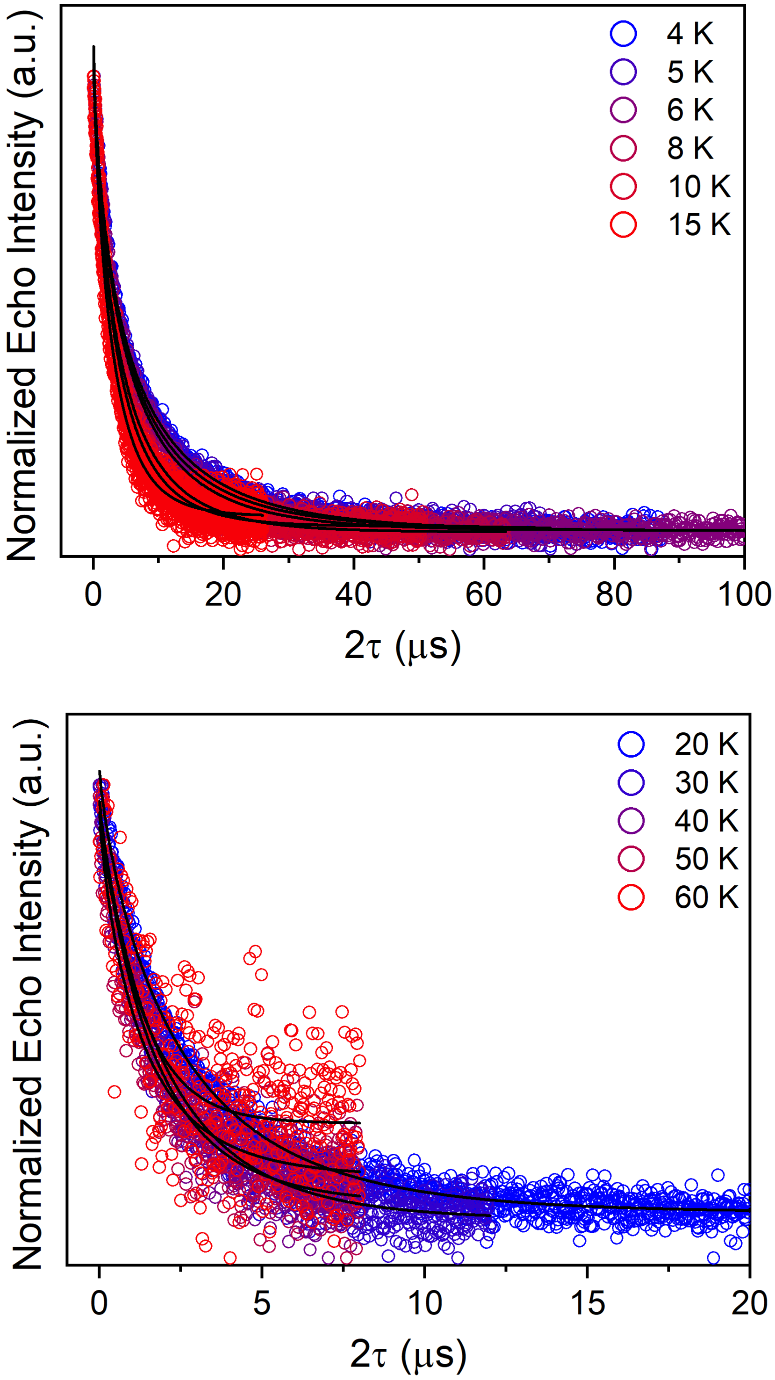

Fig. S14. Selected variable temperature Hahn echo decay curve (color traces) and fits (black traces) for **2** in d^{14}-*o*-terphenyl at 120 GHz.

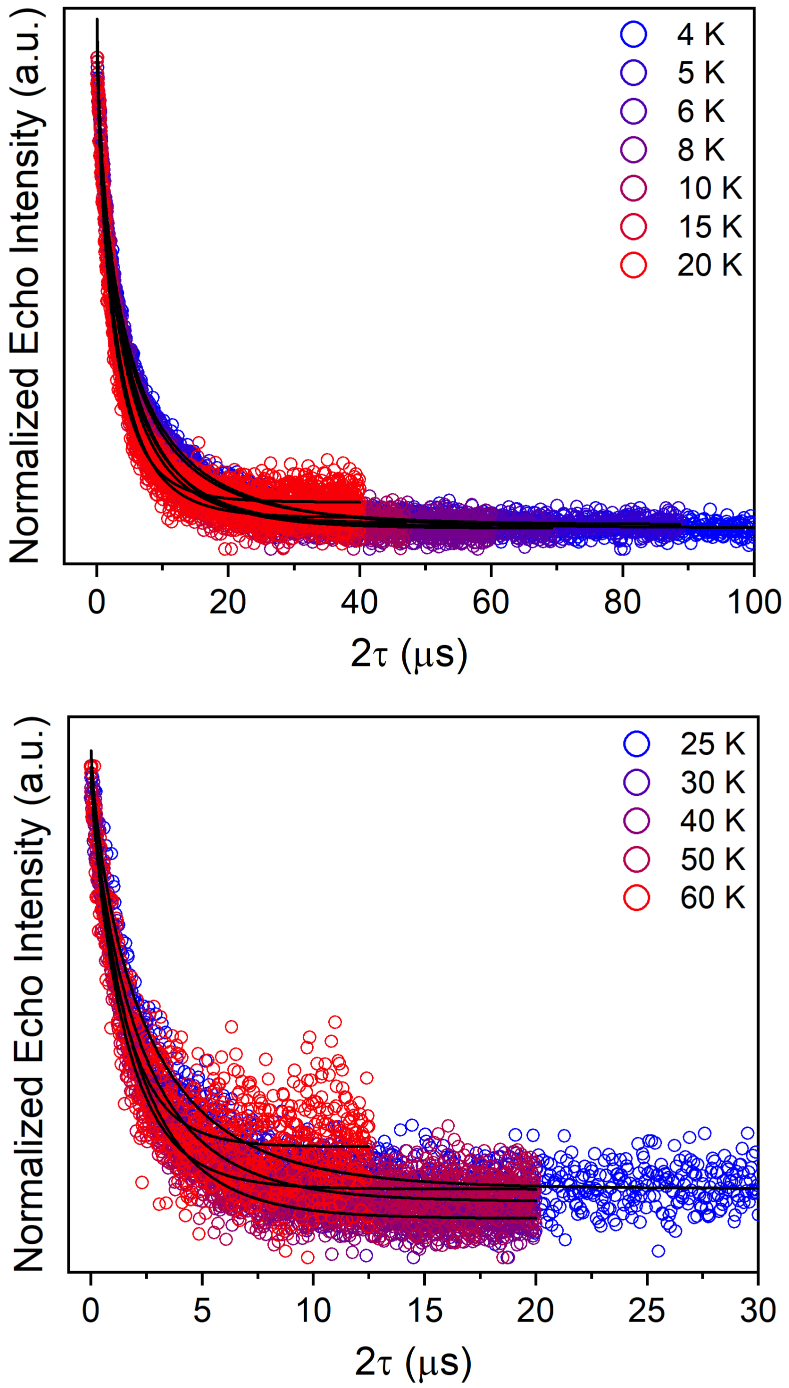

Fig. S15. Selected variable temperature Hahn echo decay curve (color traces) and fits (black traces) for **3** in d^{14}-*o*-terphenyl at 120 GHz.

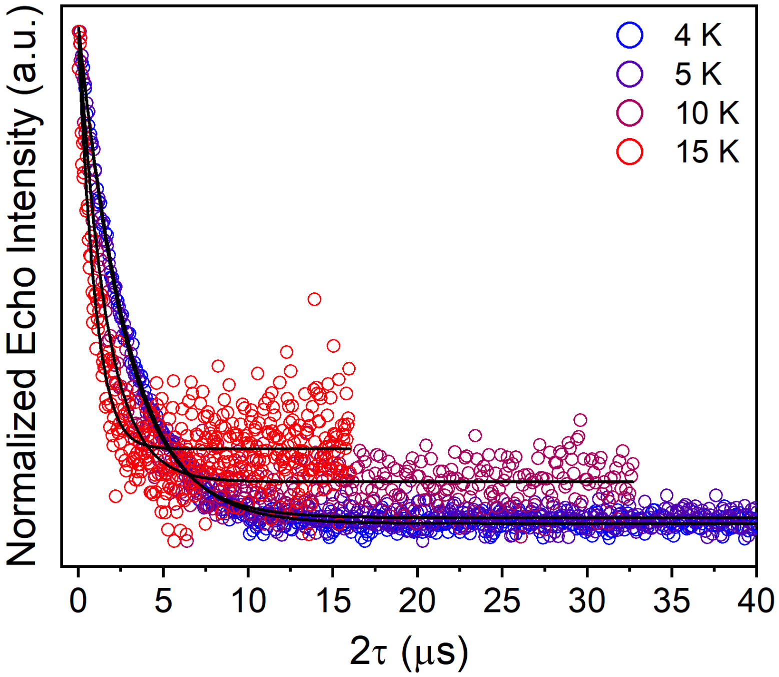

Fig. S16. Selected variable temperature Hahn echo decay curve (color traces) and fits (black traces) for **4** in *d*[14]-*o*-terphenyl at 120 GHz.

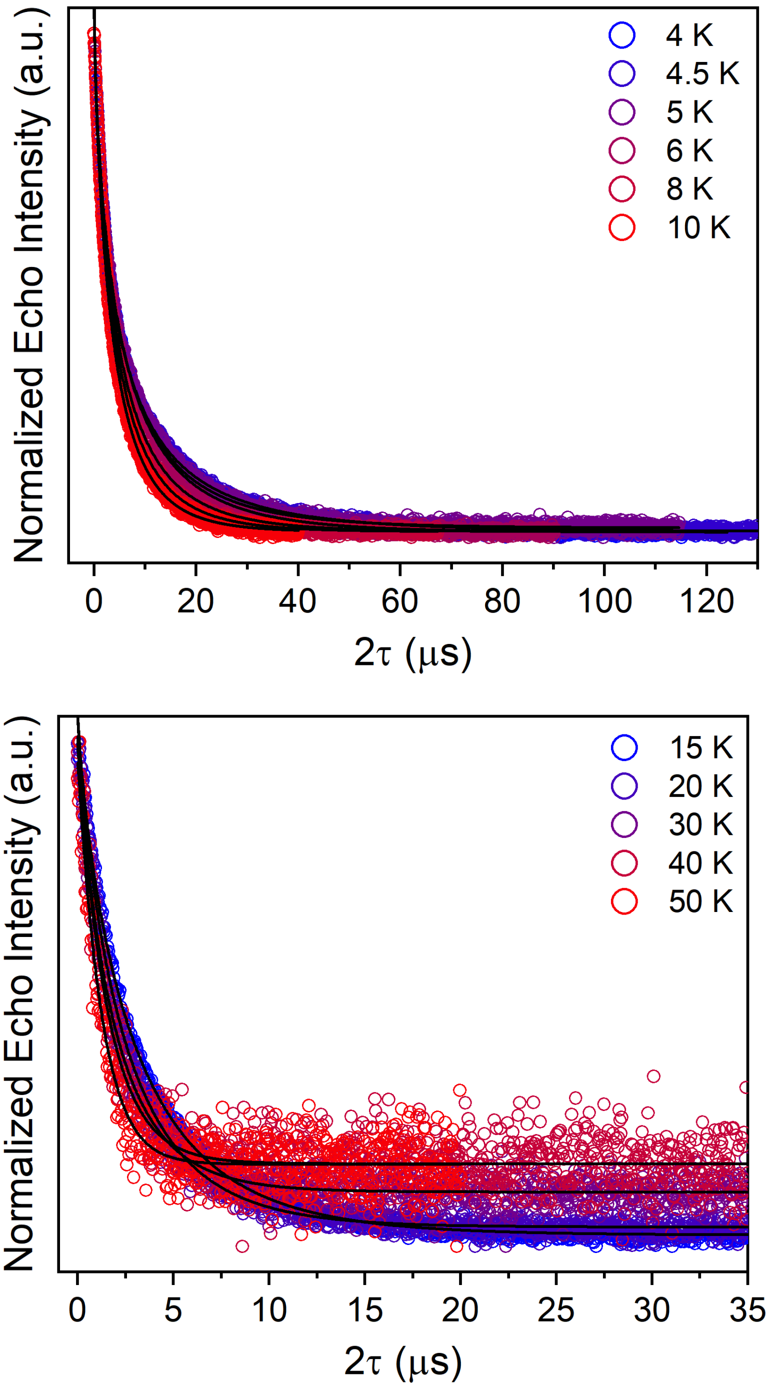

Fig. S17. Selected variable temperature Hahn echo decay curve (color traces) and fits (black traces) for **5** in d^{14}-*o*-terphenyl at 120 GHz.

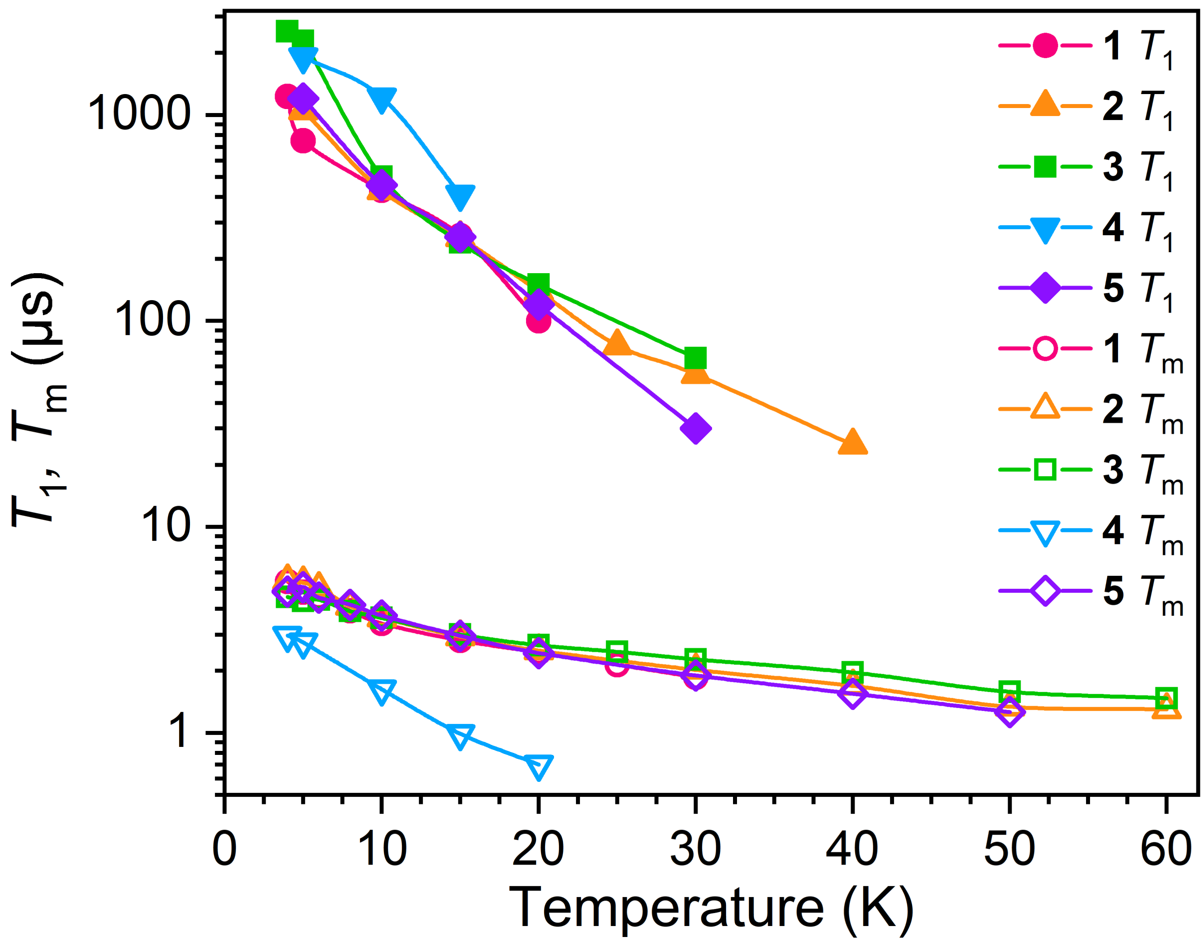

Figure S18. Variable temperature T_1 and T_m plot for **1**–**5**.

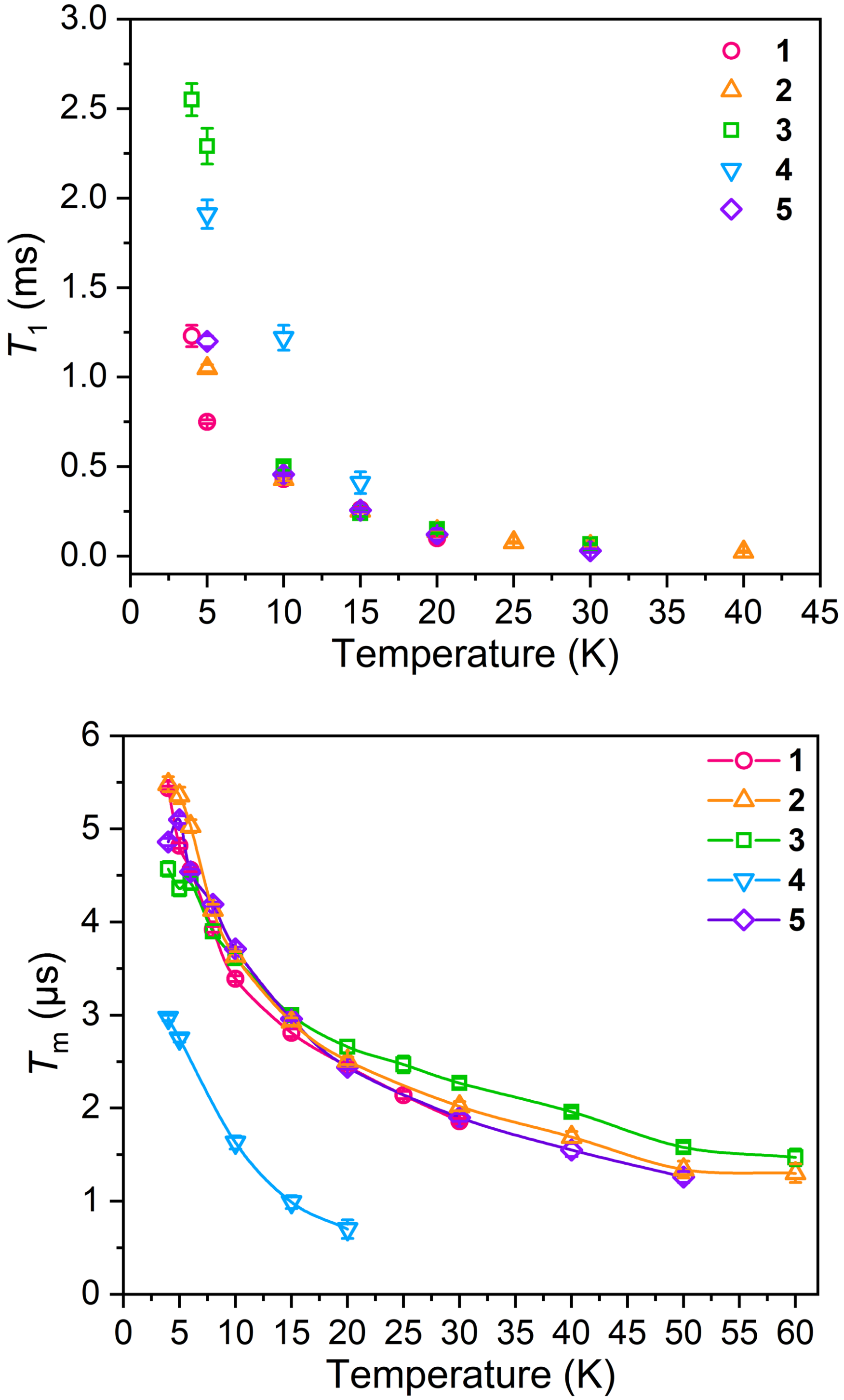

Figure S19. Variable temperature T_1 (**top**) plot for **1**–**5** with the error in T_1 shown. Variable temperature T_m (**bottom**) plot for **1**–**5** with error in T_m shown.

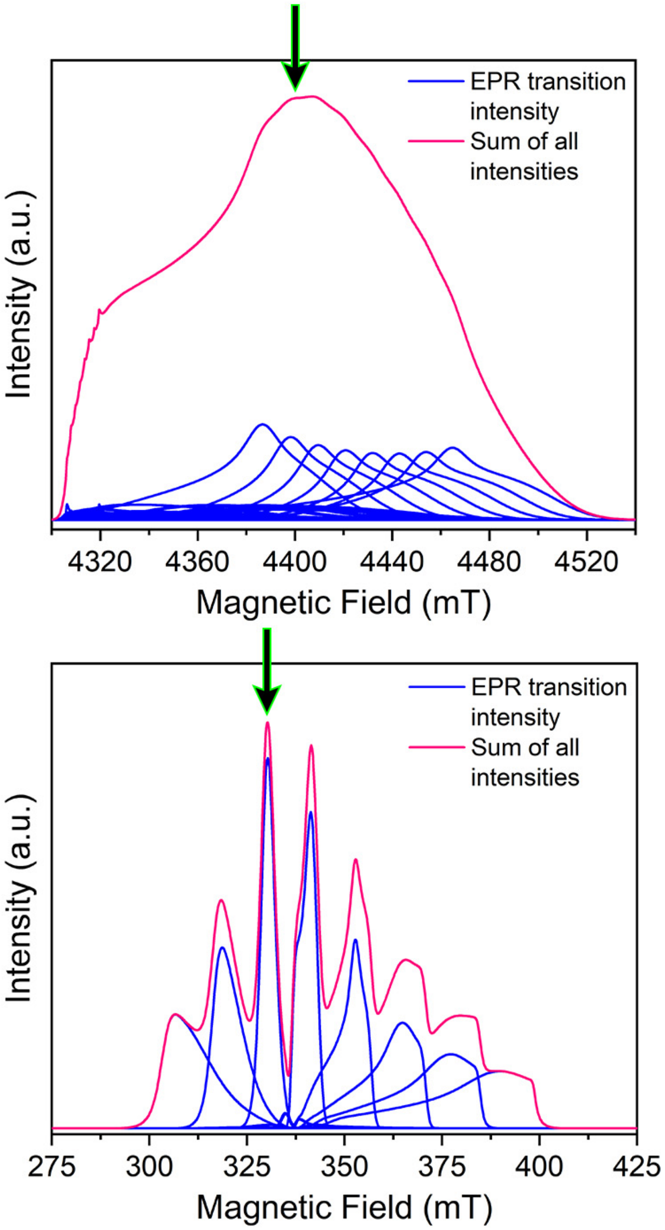

Figure S20. Comparison of probed transitions at X-band and 120 GHz. The glowing black arrow indicates the field of analysis. **Top:** Individual transitions that combine to yield the X-band spectrum. **Bottom:** Separate transitions at 120 GHz for **1** using the *g* and A values listed in Table S5. Transitions were simulated using Easyspin. The pink lines in both plots represent the sums of the intensities of all the simulated transitions for that frequency.

References.

1. Cooper, S. R.; Koh, Y. B.; Raymond, K. N. Synthetic, Structural, and Physical Studies of Bis(Triethylammonium) Tris(Catecholato)Vanadate(IV), Potassium Bis(Catecholato)Oxovanadate(IV), and Potassium Tris(Catecholato)Vanadate(III). *J. Am. Chem. Soc.* **1982**, *104* (19), 5092–5102.
2. Bryant, B. E.; Fernelius, W. C.; Busch, D. H.; Stoufer, R. C.; Stratton, W. Vanadium(IV) Oxy(Acetylacetonate). In *Inorganic Syntheses*; 1957; Vol. 5, pp 113–116.
3. Saikia, B.; Borah, P.; Chandra Barua, N. H_2O_2 in WEB: A Highly Efficient Catalyst System for the Dakin Reaction. *Green Chem.* **2015**, *17* (9), 4533–4536.
4. Kohn, M. Bromination of Catechol. *J. Am. Chem. Soc.* **1951**, *73* (1), 480–480.
5. Milsmann, C.; Levina, A.; Harris, H. H.; Foran, G. J.; Turner, P.; Lay, P. A. Charge Distribution in Chromium and Vanadium Catecholato Complexes: X-Ray Absorption Spectroscopic and Computational Studies. *Inorg. Chem.* **2006**, *45* (12), 4743–4754.
6. Sheldrick, G. M. Program for Empirical Absorption Correction of Area Detector Data. *SADABS* **1996**.
7. Sheldrick, G. M. SHELXT – Integrated Space-Group and Crystal-Structure Determination. *Acta Crystallogr. Sect. Found. Adv.* **2015**, *71* (1), 3–8.
8. Sheldrick, G. M. Crystal Structure Refinement with SHELXL. *Acta Crystallogr. Sect. C Struct. Chem.* **2015**, *71* (1), 3–8.
9. Sheldrick, G. M. A Short History of SHELX. *Acta Crystallogr. A* **2008**, *64* (1), 112–122.
10. Stoll, S.; Schweiger, A. EasySpin, a Comprehensive Software Package for Spectral Simulation and Analysis in EPR. *J. Magn. Reson.* **2006**, *178* (1), 42–55.
11. MagLab website: https://nationalmaglab.org/
12. Van Tol, J.; Brunel, L.-C.; Wylde, R. J. A Quasioptical Transient Electron Spin Resonance Spectrometer Operating at 120 and 240 GHz. *Rev. Sci. Instrum.* **2005**, *76* (7), 074101.
13. Morley, G. W.; Brunel, L.-C.; van Tol, J. A Multifrequency High-Field Pulsed Electron Paramagnetic Resonance/Electron-Nuclear Double Resonance Spectrometer. *Rev. Sci. Instrum.* **2008**, *79* (6), 064703.
14. *Matlab*; The MathWorks Inc.: Natick, MA, 2018.
15. *Origin*; OriginLab: Northampton, MA, 2018.
16. Lin, C.-Y.; Ngendahimana, T.; Eaton, G. R.; Eaton, S. S.; Zadrozny, J. M. Counterion influence on dynamic spin properties in a V(IV) complex. *Chem. Sci.* **2019**, *10* (2), 548–555.
